# Antimicrobial use and production system shape the fecal, environmental, and slurry resistomes of pig farms

**DOI:** 10.1186/s40168-020-00941-7

**Published:** 2020-11-19

**Authors:** Oscar Mencía-Ares, Raúl Cabrera-Rubio, José Francisco Cobo-Díaz, Avelino Álvarez-Ordóñez, Manuel Gómez-García, Héctor Puente, Paul D. Cotter, Fiona Crispie, Ana Carvajal, Pedro Rubio, Héctor Argüello

**Affiliations:** 1 Department of Animal Health, Faculty of Veterinary, Universidad de León, León, Spain; 2 Teagasc Food Research Centre, Moorepark, Fermoy, Co. Cork, Ireland; 3 APC Microbiome Institute, University College Cork, Co. Cork, Ireland; 4 Department of Food Hygiene and Technology, Faculty of Veterinary, Universidad de León, León, Spain; 5 Institute of Food Science and Technology, Universidad de León, León, Spain; 6 VistaMilk SFI Research Centre, Fermoy, Co. Cork, Ireland

**Keywords:** Antimicrobial resistance, Feces, Farm environment, Mobilome, One health, Sustainable farming, Swine

## Abstract

**Background:**

The global threat of antimicrobial resistance (AMR) is a One Health problem impacted by antimicrobial use (AMU) for human and livestock applications. Extensive Iberian swine production is based on a more sustainable and eco-friendly management system, providing an excellent opportunity to evaluate how sustained differences in AMU impact the resistome, not only in the animals but also on the farm environment. Here, we evaluate the resistome footprint of an extensive pig farming system, maintained for decades, as compared to that of industrialized intensive pig farming by analyzing 105 fecal, environmental and slurry metagenomes from 38 farms.

**Results:**

Our results evidence a significantly higher abundance of antimicrobial resistance genes (ARGs) on intensive farms and a link between AMU and AMR to certain antimicrobial classes. We observed differences in the resistome across sample types, with a higher richness and dispersion of ARGs within environmental samples than on those from feces or slurry. Indeed, a deeper analysis revealed that differences among the three sample types were defined by taxa-ARGs associations. Interestingly, mobilome analyses revealed that the observed AMR differences between intensive and extensive farms could be linked to differences in the abundance of mobile genetic elements (MGEs). Thus, while there were no differences in the abundance of chromosomal-associated ARGs between intensive and extensive herds, a significantly higher abundance of integrons in the environment and plasmids, regardless of the sample type, was detected on intensive farms.

**Conclusions:**

Overall, this study shows how AMU, production system, and sample type influence, mainly through MGEs, the profile and dispersion of ARGs in pig production.

Video Abstract

**Supplementary Information:**

The online version contains supplementary material available at 10.1186/s40168-020-00941-7.

## Background

Antimicrobial resistance (AMR) is one of the largest threats to global health and food security [1, 2]. Antimicrobial use (AMU) in human medicine is an important factor, but it is also widely recognized that the use of antimicrobials in food-producing animals contributes to the burden of AMR in human health [3]. The frequent AMU to treat or prevent infections in livestock, mainly through prophylactic and metaphylactic administration in feed or water, together with the misuse of antimicrobials as growth promoters in certain countries, have facilitated the selection and spread of AMR bacteria [4–6].

The pig industry is the most extensive agricultural user of antimicrobials in the European Union [7, 8]. Monitoring of indicator and zoonotic bacteria on pig farms reveals a frequent detection of AMR [9] and the presence of certain antimicrobial resistance genes (ARGs) of critical importance, such as *bla*_CTX-M_, *mecA*, or *mcr* [10–12]. Metagenomic approaches complement traditional AMR surveillance systems by characterizing the total pool of ARGs, including those on mobile genetic elements (MGEs), in the whole microbial community [13–15].

Recent studies describing the fecal resistome in pigs have suggested a direct link between the resistome (the collection of all resistance genes in a microbiome) and AMU or the country in which the farm was located [16, 17]. Associations between animal genetics, age or diet with microbiome composition and, therefore, with its resistome, have also been uncovered [16]. So far, pig resistome studies have been performed in industrialized intensive swine herds [18, 19]. The traditional extensive system, mainly associated with the Iberian pig breed (*Sus scrofa domesticus*) in Spain, is defined by eco-friendly and sustainable husbandry practices, including the constrained use of antimicrobials [20], which has been maintained for decades. Thus, this extensive production system offers an ideal means to study how sustained differences in AMU have impacted the resistome of animals and the farm environment.

To address these knowledge gaps, this study uses a metagenomic approach to characterize structural, qualitative, and quantitative differences in the resistome, and its associated mobilome, from 467 pooled fecal, environmental, and slurry samples from 38 pig farms.

## Results

### Resistome alpha diversity and richness of ARGs

**Fig. 1.**
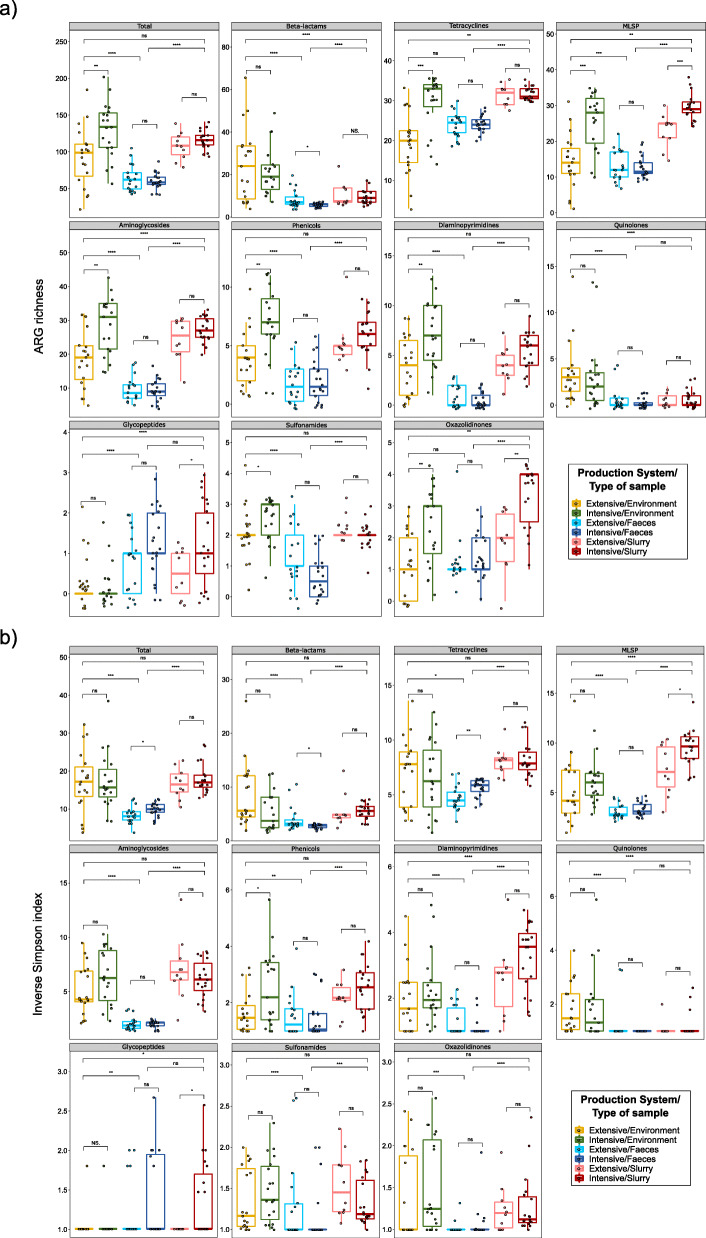
Alpha diversity of different antimicrobial resistance (AMR) classes measured by antimicrobial resistance genes richness (ARGs) (**a**) and Inverse Simpson (**b**) indexes. These indexes were calculated from the counts per million matrix and represented as boxplots. Each sample is represented by a dot with horizontal jitter for visibility. The horizontal box lines represent the first quartile, the median, and the third quartile. Whiskers include the range of points within the 1.5 interquartile range. The differences per sample type and per production system within each sample type were evaluated with the Wilcoxon signed-rank test. *n* = 105 metagenomes from 38 independent farms. Nineteen metagenomes per sample type per production system were used, with the exception of extensive-slurry (*n* = 9). MLSP refers to the macrolides-lincosamides-streptogramins-pleuromutilins AMR class

One hundred five metagenomes from 467 pooled fecal, environmental, and slurry samples from 19 intensive pig farms and 19 extensive swine farms were sequenced. The average number of reads obtained per sample was 8.1 million (range 5.3 million–9.8 million). An average of 0.1% of these reads were assigned to ARGs (range 0.004–0.35%). The alpha diversity of the resistome was calculated for each sample (Fig. 1; see Additional file 1: Figure S1). Inverse Simpson and ARG richness indexes showed that the total ARGs diversity was significantly lower in feces, both from intensive and extensive farms, than in farm environments and slurry samples. A similar result was observed for almost all AMR classes, when analyzed individually (Fig. 1).

More importantly, analyses by production system revealed a significantly higher ARG richness on the environmental samples from intensive farms than in those from extensive herds. This applied to both total ARGs (*p* < 0.01) and for most AMR classes (Fig. 1a). Furthermore, the Inverse Simpson index showed a significantly higher diversity for tetracycline ARGs within the fecal microbiomes from intensive farms as compared to those from extensive herds (*p* < 0.01) (Fig. 1b).

### Resistome beta diversity analysis

**Fig. 2:**
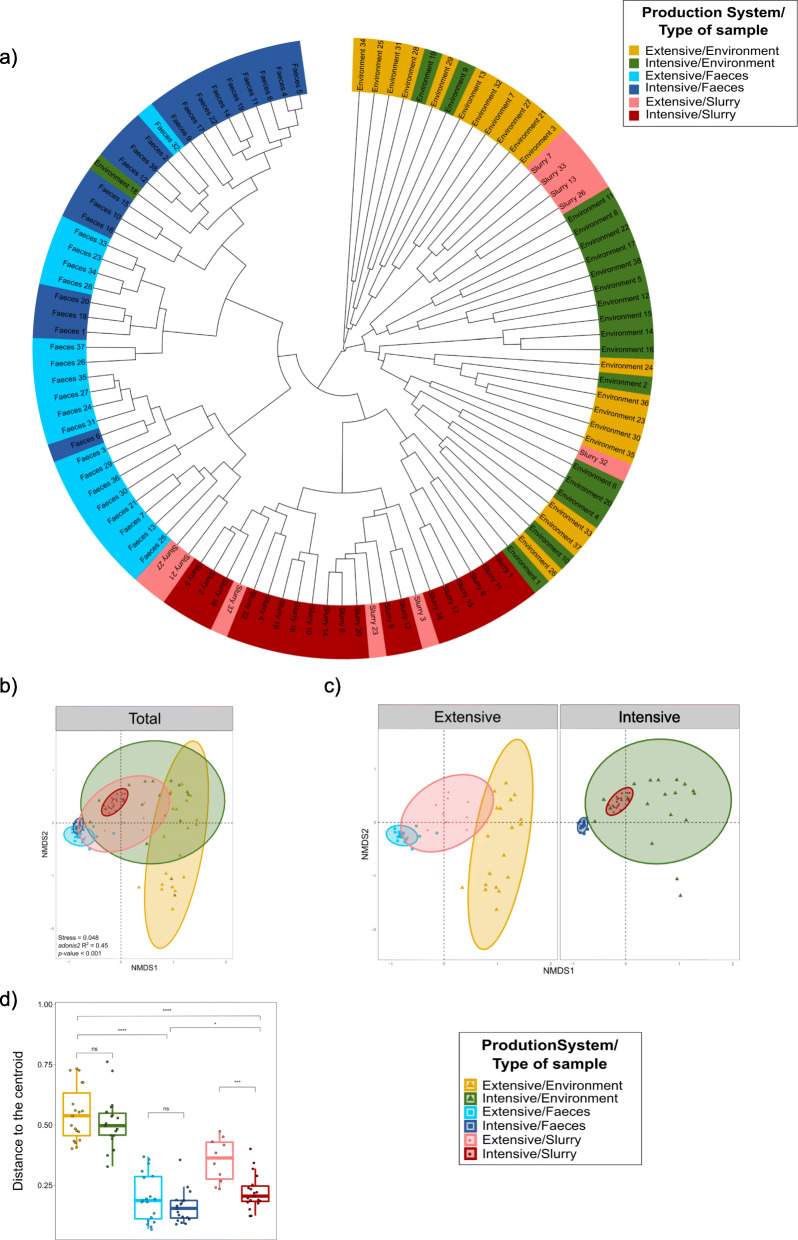
Resistome structure in samples from three sample types on intensive and extensive farms. **a** Dendrogram showing the complete linkage clustering of Bray-Curtis dissimilarities among intensive and extensive pig farms per sample type. **b** Two-dimension non-metric multidimensional scaling (NMDS) based on Bray-Curtis dissimilarities. Subsampling was carried out by the three types of samples within each production system prior to performing ordination analysis and PERMANOVA. The centroid of each ellipse represents the group mean, and the shape was defined by the covariance within each group. **c** NMDS resulting from the division of the previous analysis by the two production systems to observe clearer differences. **d** Distance to the centroid for the evaluation of homogeneity of variances within each group. *n* = 105 metagenomes from 38 independent farms. Nineteen metagenomes per production system per sample type were used, with the exception of extensive-slurry (*n* = 9).

A comparison of the beta diversity of ARGs using the Bray-Curtis dissimilarity index revealed the combined influence of sample type and production system (adonis2, *p* < 0.001) in the clustering and ordination of samples (Fig. 2a; Fig. 2b). However, while the type of sample explained 37.9% of the variation, the production system was of lesser importance (5.7%).

On intensive farms, the ordination showed that slurry samples represented a tightly clustered group of samples located within a more heterogeneous cluster of environmental samples, with fecal samples grouped closely nearby. In extensive herds, the slurry resistome was more highly dispersed, and was located between clusters representing farm environment and pig feces samples (Fig. 2c). The effect of the production system (adonis2, *p <* 0.001) explained 36.9% of the variation in feces, 17.8% in slurry and 8.8% in the environment. Beta dispersion varied significantly among sample types, with higher dispersion being observed in environmental samples; while between production systems there were only significant differences in the dispersal of slurry samples (*p* < 0.001) (Fig. 2d).

Specific ordination effects among ARGs associated with resistance to beta-lactams, tetracyclines, aminoglycosides, and macrolides-lincosamides-streptogramins-pleuromutilins (MLSP) were identified (see Additional file 2: Figure S2 and Additional file 3). We observed that ordination patterns were similar to those described for the total resistome, with a strong effect of the production system on the fecal resistome (i.e., 41.2% of the variation (adonis2, *p* < 0.001) in ARGs related to tetracycline resistance).

### Characterization of the acquired resistome

**Fig. 3:**
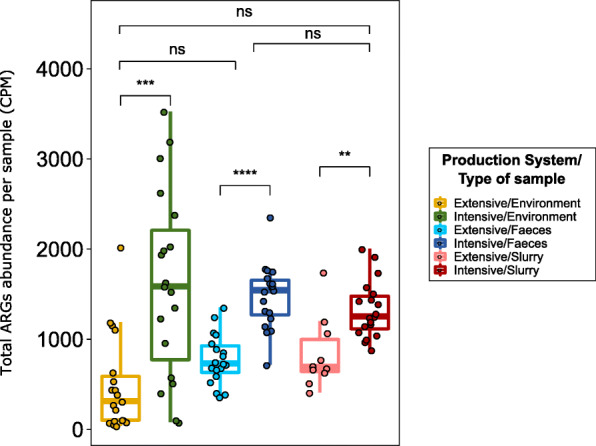
Overview of total antimicrobial resistance genes (ARGs) abundance per sample. Boxplots of the total ARGs in counts per million per sample, stratified by production system and sample type. Each sample is represented by a dot with horizontal jitter for visibility. The horizontal box lines represent the first quartile, the median, and the third quartile. Whiskers include the range of points within the 1.5 interquartile range. The differences per sample type and per production system within each sample type were evaluated with the Wilcoxon signed-rank test. *n* = 105 metagenomes from 38 independent farms. Nineteen metagenomes per sample type per production system were used, with the exception of extensive-slurry (*n* = 9)

Total ARGs abundance varied significantly (*p* < 0.01) by production system regardless of the sample type (Fig. 3). This was also evident when the abundance of ARGs from the aminoglycosides, MLSP, oxazolidinones, and tetracyclines AMR classes was analyzed (see Additional file 4: Figure S3). We ranked samples by ARG abundance and observed that seven intensive farm environments were among the top 10 samples, all showing over 2000 counts per million (CPM) of total ARG abundance. In contrast, ARG abundances on extensive farm environments were among the lowest observed, with seven of these samples showing under 200 CPM (Fig. 3). While ARGs linked to beta-lactam resistance were significantly more abundant (*p* < 0.01) in feces recovered from intensive farms than in those from extensive farms (see Additional file 4: Figure S3), their diversity was significantly lower in fecal samples from intensive farms (*p* < 0.05) (Fig. 1a), indicating a larger number of more homogeneous beta-lactam ARGs in feces from intensive farms.

**Fig. 4.**
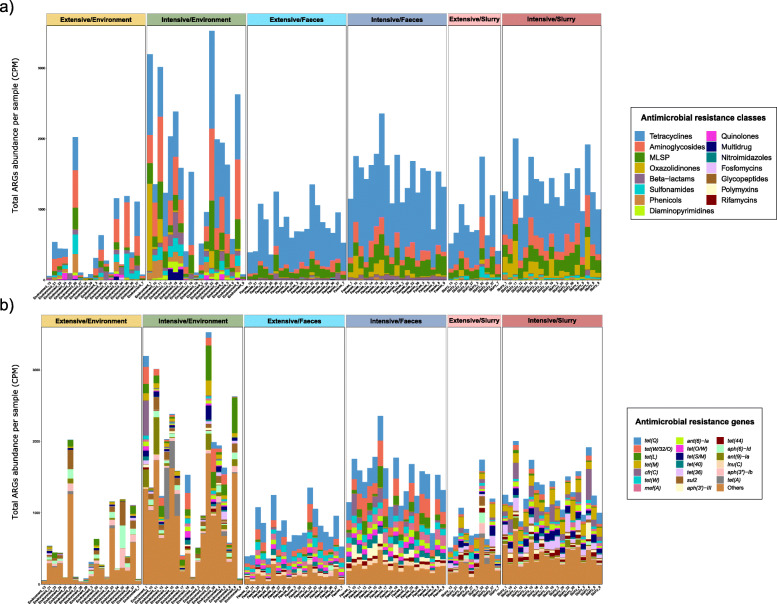
Distribution of antimicrobial resistance genes (ARGs) abundance and composition. **a** Stacked bar plot of total ARGs abundance per antimicrobial class (colors), per sample (*x* axis). **b** Stacked bar plot of 20 most abundant ARGs (colors), per sample (*x* axis); the less abundant ARGs were grouped into “Others”. *n* = 105 metagenomes from 38 independent farms. Nineteen metagenomes per sample type per production system were used, with the exception of extensive-slurry (*n* = 9). ARGs abundance was expressed as counts per million. MLSP refers to the macrolides-lincosamides-streptogramins-pleuromutilins AMR class

The abundance of ARGs linked to 15 different AMR classes was stacked to identify trends across production systems and sample types (Fig. 4a). Analyses by production system showed similar patterns of AMR class distribution for slurry and feces, but with lower ARG abundance on extensive farms, as described previously. The environmental samples showed less consistent patterns, with a higher heterogeneity of AMR classes found, both on intensive and extensive farms. The tetracycline class was predominant, particularly in feces and slurry, followed by the aminoglycosides, MLSP, and oxazolidinones classes. The sulfonamide class was significantly more abundant in slurry than in fecal samples (*p* < 0.001), while the opposite trend was found for the beta-lactam class (*p* < 0.001).

The 20 most abundant ARGs were represented to characterize AMR abundance at gene level (Fig. 4b). This ARG distribution was impacted by the type of sample. The tetracycline ARGs *tet(Q)* and *tet(W/32/O)* were predominant in feces (*p* < 0.001), while *tet(M)* and *tet(36)* were the most abundant tetracycline ARGs in slurry (*p* < 0.001). Again, the environmental samples showed a higher ARGs heterogenicity, especially on intensive farms. The high abundance of the oxazolidinone *cfr(C)* gene on samples from certain intensive farms was remarkable and was significantly less abundant among extensive herds (*p* < 0.001). All significant differences (*p <* 0.05) across production systems and sample types are shown at AMR class and ARGs level in an additional file (see Additional file 5).

### Bacterial microbiome composition and its association with the resistome

To evaluate the degree to which the bacterial composition determined the resistome, Procrustes analyses were performed. We observed a significant correlation between the resistome and the bacterial microbiome composition (*p* < 0.001; correlation = 0.71), demonstrating that similar taxonomic compositions tended to have a similar antimicrobial resistance profile (see Additional file 6: Figure S4). Interestingly, sample type and production system impacted significantly (*p* < 0.001) on this association, with stronger association on intensive farms (correlation = 0.81) than in extensive herds (correlation = 0.72) and in slurry (correlation = 0.70) than environmental (correlation = 0.64) and fecal (correlation = 0.53) samples. Further details in bacterial microbiome data can be accessed in additional files (see Additional file 7: Supplementary information and Additional file 8: Figure S5).

### Taxonomic assignment of ARGs

Ninety-four percent of the obtained ARG-reads were assigned to ARG-containing contigs. These ARG-containing contigs were predicted to belong to 120 different bacterial families, with *Streptococcaceae*, *Bacteriodaceae*, *Peptostreptococcaceae*, *Staphylococcaceae*, *Enterobacteriaceae*, *Moraxellaceae*, *Lactobacillaceae*, *Bacillaceae*, *Enterococcaceae*, and *Clostridiaceae* accounting for 58.5% of the total ARGs abundance and 75.8% of all assigned ARGs. Most of these families were among the most abundant taxa on these farms, with exemptions such as *Peptostreptococcaceae* and *Enterococcaceae*, whose proportion in the total bacterial microbiome was much smaller. In contrast, despite their high abundance, *Pseudomonadaceae*, *Prevotellaceae*, or *Flavobacteriaceae* families did not contribute remarkably to the resistome composition.

**Fig. 5.**
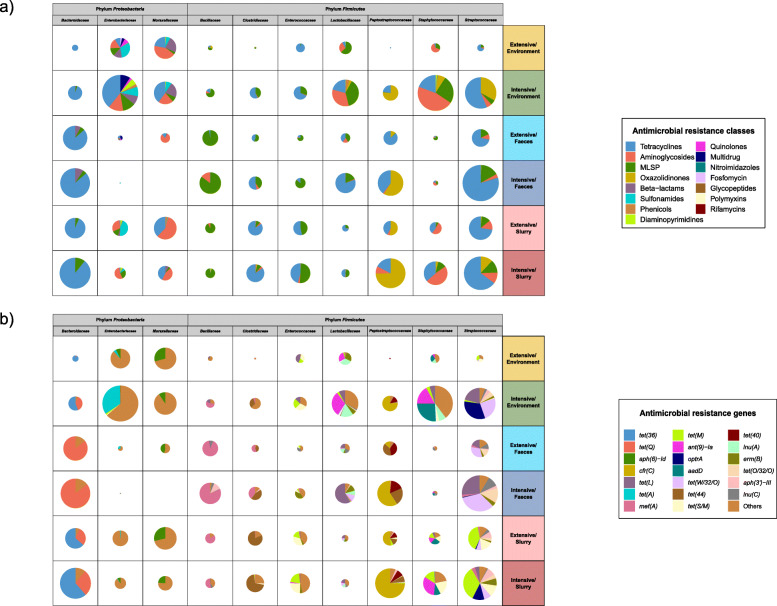
Taxonomical assignment of the resistome at family level. The abundance of antimicrobial resistance genes (ARGs) taxonomically assigned was expressed in counts per million, selecting the 10 most abundant taxonomical families harboring ARGs. **a** Pie chart distribution of total AMR abundance per antimicrobial class (colors), per taxonomical family, production system and sample type; the size of each pie chart is proportional to the ARGs abundance within each group. **b** Pie chart distribution of the 20 most abundant ARGs in the 10 most abundant taxonomical families harboring ARGs (colors), per taxonomical family, production system and sample type; the less abundant ARGs were grouped into “others”. *n* = 105 metagenomes from 38 independent farms. Nineteen metagenomes per sample type per production system were used, with the exception of extensive-slurry (*n* = 9). MLSP refers to the macrolides-lincosamides-streptogramins-pleuromutilins AMR class

We further investigated the 10 most abundant families with assigned ARGs from the main AMR classes (Fig. 5a). The distribution of AMR-encoding taxa at family level varied across and within families by production system and type of sample. Thus, in *Bacteroidaceae* and *Streptococcaceae*, ARGs from the tetracycline AMR class were predominant, while those from the MLSP and oxazolidinone AMR classes were the most abundant in *Bacillaceae* and *Peptostreptococcaceae*, respectively. The impact of the production systems (*p* < 0.05) on the abundance of ARGs assigned to *Enterobacteriaceae*, *Staphylococcaceae*, and *Streptococcaceae* was remarkable, with a higher abundance on intensive farms, particularly in environmental samples.

Similar analyses by the 20 most abundant ARGs assigned to these 10 taxonomical families (Fig. 5b) revealed a gene-specific taxonomical association of *tet* ARGs, a result which helps to explain their heterogeneous distribution among sample types shown in Fig. 4b. More specifically, the *tet(Q)* gene was associated with members of the *Bacteroidaceae* family, particularly in feces, while other tetracycline ARGs, such as *tet(L)* and *tet(M)*, were mainly linked to families belonging to the *Firmicutes* phylum, such as *Streptococcaceae*, *Staphylococcaceae*, *Lactobacillaceae*, or *Enterococcaceae*. The oxazolidinone ARG *cfr(C)* was predominantly associated with the *Peptostreptococcaceae*, while *optr(A)* was most abundant in *Streptococcaceae*, both showing a significantly higher abundance on intensive farms (*p* < 0.001). Further details of differences by production system and sample type in taxonomical assignation of ARGs, both at AMR class level and individual ARG level, are summarized in an additional file (see Additional file 9).

### Characterization of the AMR mobilome

**Fig. 6:**
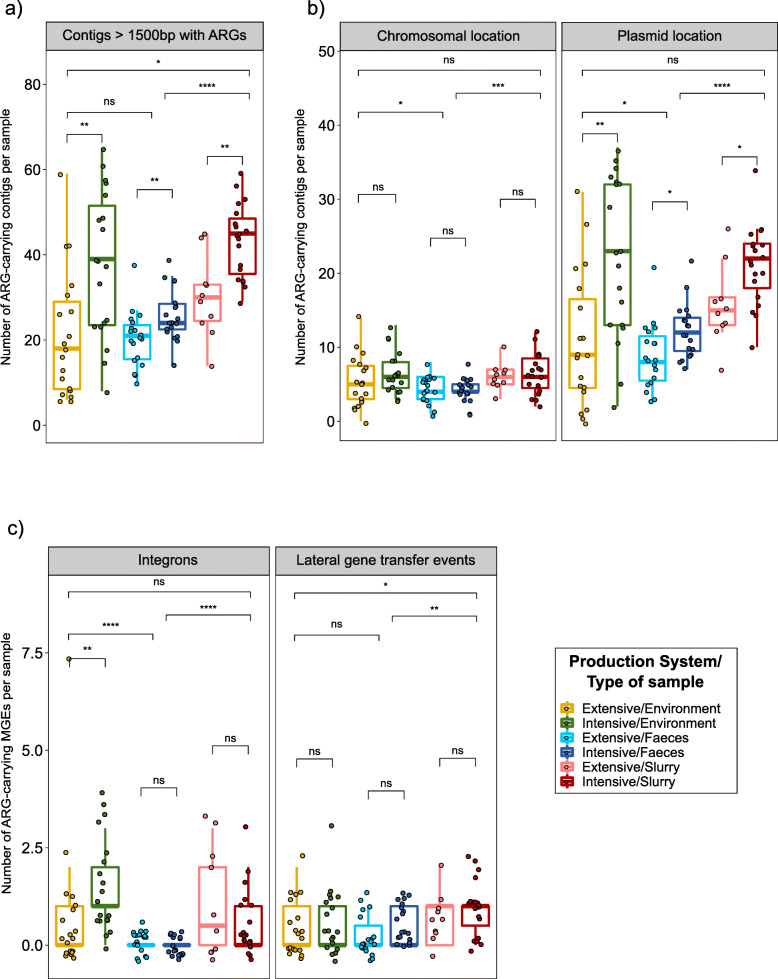
Antimicrobial resistance mobilome characterization. **a** Boxplot of contigs with more than 1500bp carrying antimicrobial resistance genes (ARGs). **b** Boxplots of chromosomal or plasmid location of ARGs containing contigs with PlasFlow. **c** Boxplots of integrons and lateral gene transfer events involving ARGs detected in contigs with Integron_Finder and WAAFLE, respectively. Each sample is represented by a dot with horizontal jitter for visibility. The horizontal box lines represent the first quartile, the median, and the third quartile. Whiskers include the range of points within the 1.5 interquartile range. The differences per sample type and per production system within each sample type were evaluated with the Wilcoxon signed-rank test. *n* = 105 metagenomes from 38 independent farms. Nineteen metagenomes per sample type per production system were used, with the exception of extensive-slurry (*n* = 9)

We evaluated the presence of ARGs on 678,111 contigs that contain MGEs in all samples after metagenomics assembly. Thereof, we identified 3130 contigs (0.5%) larger than 1500 base pairs (bp) that contained ARGs. The number of these ARG-containing contigs was significantly higher on samples from intensive farms than on those from extensive herds (*p* < 0.01), regardless of the sample type (Fig. 6a). These ARG-containing contigs were predicted to be mainly, but not exclusively, regions of plasmids (Fig. 6b). Notably, while a significantly higher number of ARGs were located in plasmids on samples from intensive farms (*p* < 0.05), no significant differences were observed between production systems in the abundance of ARGs of chromosomal location (Fig. 6b). Correlation analyses revealed a significant association (*p* < 0.05) between the abundance of plasmids carrying ARGs and tetracycline use (*r*_*s*_ = 0.41) and total AMU (*r*_*s*_ = 0.51), suggesting that the resistome is associated with AMU. This pattern was also apparent for environmental and fecal samples (*r*_*s*_ ≥ 0.47), but not for slurry samples.

The abundance of integrons was significantly higher within environments from intensive than on extensive farms (*p* < 0.01), while integrons were relatively uncommon in fecal samples (Fig. 6c). A total of 68 integrons were characterized carrying 144 ARGs, which clustered in 39 different groups, 2 of which contained 23.9% of the identified integrons. The aminoglycoside ARGs *aadA* (representing 61.1% of the ARGs found in integrons), the trimethoprim ARGs *dfrA* (19.4%), or both (31.3% of the integrons) were frequently contained within these regions. Lateral gene transfer (LGT) events were significantly more frequent in slurry than in fecal or environmental samples (*p* < 0.05), but no differences were observed between the two production systems (Figure 6c). We detected 57 LGT events involving 59 ARGs, among which tetracycline ARGs were the most abundant (59.6% of the LGT events), with bacteria from the class *Clostridia* as the main donor, particularly for the *tet(W/32/O)* gene. ARGs linked to resistance to MLSP were detected in 21.1% of the LGT events with the *Bacteroidia* class being the main donor bacteria in this instance. Further information on the LGT events and integrons identified, including the ARGs involved and their clustering, are available in an additional file (see Additional file 10).

### The resistome is associated with AMU

Correlation of global ARGs abundance data and total AMU revealed a significant association (*p* < 0.05) between AMU and the abundance of ARGs from the aminoglycoside (*r*_*s*_ = 0.52), tetracycline (*r*_*s*_ = 0.55), MLSP (*r*_*s*_ = 0.58), and oxazolidinone (*r*_*s*_ = 0.68) AMR classes (see Additional file 11: Figure S6A). Analysis on AMU from the perspective of antimicrobial class level yielded significant correlations between the abundance of ARGs from the oxazolidinone AMR class and the use of phenicols (*r*_*s*_ = 0.45), macrolides-lincosamides-pleuromutilins (MLP) (*r*_*s*_ = 0.60), or tetracyclines (*r*_*s*_ = 0.45).

Similar association patterns were observed for fecal and environmental resistomes when analyzed individually (*r*_*s*_ ≥ 0.43, see Additional file 11: Figure S6B and Figure S6C)). Indeed, the impact of AMU on the abundance of ARGs was even more marked (*r*_*s*_ ≥ 0.58) in fecal samples. In both sample types, MLP and phenicols consumption was positively associated (*r*_*s*_ ≥ 0.43) with the abundance of ARGs from most AMR classes. Although only a few positive correlations were observed for slurry samples, the general patterns were consistent with the results obtained for fecal and environmental samples (see Additional file 11: Figure S6D). No remarkable associations were observed for other antimicrobial classes, including the beta-lactams, despite their high use on these farms.

## Discussion

The characterization of farm metagenomes provided an integral overview of differences in the resistome of animals and of the farm environment across two swine production systems. The farm resistomes were defined by a combination of three factors: production system, sample type, and antimicrobial consumption at farm level.

Extensive Iberian pig production generates high quality cured products within a particular rearing system, which includes differentiating factors such as outdoor farming in oak fields, lower animal density, and/or the compulsory slaughter at age 14 months [21], instead of at age 6–8 months in the case of pigs reared on intensive farms. This extensive approach translates to greater health and thus a lower AMU [20]. The lower ARG abundance detected in samples collected from these extensive farms, regardless of the sample type (i.e., environments, feces or slurry), is likely primarily linked to the significantly lower AMU on these farms, which is the main factor driving the rise and spread of ARGs in animal fecal microbiomes [16, 17, 22–24]. As noted, additional factors, such as feeding regime, husbandry practices, or farm environments, which have been previously suggested as having an influence on the resistome composition in cattle [25, 26], might also contribute to the differences observed in this study.

Slurry and farm environments may play an important role in the spread and on farm re-circulation of ARGs and AMR bacteria. We disclosed structural differences between the resistome of these two sample types and that of pig feces. Indeed, both slurry and environmental samples exhibited a higher ARG richness and beta dispersion when compared to the resistome of fecal samples, regardless of the AMR class and even on intensive farms, where feces and farm environments are in closer and continuous contact. This agrees with results from previous studies in cattle [27] and reflects that within farm environments there are different micro-ecosystems with a wide range of microbes, including indigenous microbiota [28], and ARGs present. In addition, the strong effect shown by sample type in the resistome was also observed for the bacterial microbiome composition, with, for instance, the dominance of *Proteobacteria* in environmental samples, in contrast with a more diverse taxonomy in feces and slurry, with the predominance of members of the *Firmicutes* phylum. These findings suggested an association between the resistome and the microbiome, which was further supported by Procrustes analyses, evidencing that changes in the environment and slurry resistomes were linked to shifts in the microbial populations dominating these niches, as it has been recently reported on pig farms [29].

Some major differences between the resistome of feces and farm environments were found, for instance, in the abundance of ARGs assigned to *Enterobacteriaceae*. This family, which includes bacteria of relevance for public health [30], was a sub-dominant taxa in feces due to the relatively small proportion of members of the *Proteobacteria* phylum in fattening pig feces [31, 32], but represented a major group in environmental samples. In the farm environment, this family, together with certain families from the *Firmicutes* phylum, becomes the dominant taxonomic groups carrying ARGs, probably due to their aerotolerance, which would provide them a competitive advantage over other fecal-associated strict anaerobic taxa. Thus, the resistome structure on the farm environment, regardless of the production system, was determined by the high abundance of a combination of soil-associated bacteria, such as members of the *Moraxellaceae* or *Staphylococcaceae* families, together with fecal-associated facultative anaerobic bacteria, such as *Enterobacteriaceae*, *Streptococcocaeae*, or *Lactobacillaceae*.

The fecal and slurry resistomes had a similar qualitative composition at AMR class level, but clear differences were observed with respect to the specific ARGs, suggesting that the composition of the bacterial community dominating each sample type shapes the associated resistome, as previously proposed [33, 34]. Through the taxonomic assignment of ARGs to bacterial families, we confirmed that such differences by sample type were linked to a differential abundance of ARG-containing taxa. For instance, the tetracycline ARG *tet(Q)*, the most abundant gene within the most common AMR class, was almost exclusively assigned to the *Bacteroidaceae* family, which agrees with previous reports [35]. While this ARG was predominant in fecal *Bacteroidaceae*, slurry and, to a lesser extent, environmental *Bacteroidaceae* frequently carried also the *tet(36)* ARG.

We also found that the oxazolidinone-resistance genes *cfr(C)* and *optrA* were mainly associated with members of the *Peptostreptococcaceae* and *Streptococcacae* families, respectively. This agrees with previous studies describing that the *cfr(C)* gene was mainly confined to *Clostridioides difficile* [36, 37], and that the *optrA* gene was previously described in *Streptococcus* of swine origin [38–40]. In our study, both genes were mainly identified in samples from intensive herds and were associated with high consumption of phenicols and MLP at farm level. Despite the fact that oxazolidinones are not currently used in food-producing animals [41], these two ARGs confer resistance to other antimicrobial families which have been widely used on swine farms, such as phenicols in *cfr(C)*, and phenicols, lincosamides, pleuromutilins, and streptogramins A in *optrA* [42]. Altogether, these facts demonstrate that the cross-selection for AMR to last resort antimicrobials can occur on swine farms.

In our study, the most consistent associations between abundance of ARGs and AMU were observed for ARGs from the tetracyclines or MLSP AMR classes with the application of antimicrobials from these respective groups, which are frequently administered during the fattening period, in agreement with a recent study carried out by Van Gompel et al. [43]. Although we did not collect AMU data corresponding to the early stages of production, this previous study reported the absence of an association between AMU during these early stages and the resistome at the end of the fattening period. Interestingly, despite beta-lactams were one of the most frequently used antimicrobials on these farms, particularly in intensive herds, its use was not significantly associated with the abundance of ARGs. When compared to other antimicrobial classes which exhibited positive resistome-AMU correlations, we observed lower abundance of beta-lactam ARGs in MGEs. Further research is needed to establish the possible link between short-term AMU data and AMR spread through MGEs depicted in this study. Furthermore, associations observed between AMU and resistance to antimicrobials of different AMR classes suggests that ARGs are selected and enriched in the absence of exposure to the AMR class they confer resistance to. This arises through their co-selection due to the use of other antimicrobials or the enrichment of certain components of the microbiome [14].

MGEs promote the mobilization and dissemination of ARGs in bacterial communities [44–46]. A large proportion of the resistome in the present study was not only associated with MGEs, and mainly with plasmids, but also with integrons. The majority of integrons that contained ARGs carried the aminoglycoside *aadA* and trimethoprim *dfrA* ARGs, a phenomenon that was highlighted in previous surveys conducted on zoonotic and indicator bacteria on Spanish swine farms [47, 48]. Remarkably, plasmid-associated ARGs were predominantly found on intensive farms and linked to a high tetracycline consumption and total AMU. Integrons were also more abundant in samples from intensive farm environments, with a potential association with members of the *Enterobacteriaceae* family [49]. Altogether, these results demonstrate that the significant differences in ARG abundance observed between intensive and extensive production systems were mainly associated with a higher abundance of MGEs on intensive farms, probably favored by a higher AMU. The high abundance of ARGs and MGEs-associated ARGs on farm environments and slurry evidences the risk of their transmission and spread.

The metagenomic approach followed in our study could be adopted to characterize the resistome in food-producing animals in future AMR surveillance schemes through an integral analysis of the whole microbial community [50]. Using a combination of read-mapping techniques and metagenomic assembly pipelines, we could observe the actual association existing between the microbiome, mobilome, and resistome of pig farms. However, the sequencing depth can represent a limiting factor, and low abundant genes could be easily underreported [51]. Besides, the presence of particular ARGs does not necessarily mean that they will be expressed, and, therefore, discrepancies could occur with the results of phenotypic susceptibility testing. That is why Forslund et al. [24] introduced the concept “antibiotic-resistance potential”, to account for differences in gene expression and regulation that could affect phenotypic resistance.

## Conclusions

To the best of our knowledge, the current study provides the first integral analysis of the resistome on swine farms that compares two different production systems, extensive and intensive, exploring the animals, the farm environments, and slurry. A higher ARG abundance was observed in samples recovered from intensive swine farms, with higher AMU, relative to those from extensive herds. A differential distribution of ARGs was also observed among different types of samples, likely due to the dominance of different bacterial taxa in different sample types, as clearly shown by the distribution of tetracycline ARGs. Finally, the majority of identified ARGs were located on plasmids and differences in ARGs abundance among production systems were linked to a higher abundance of plasmids on intensive farms, highlighting the importance of the mobilome in the spread of ARGs on swine farms. Overall, these results show that sustainable farming practices can help reduce AMR pressure in the food chain.

## Methods

### Farms selection and sample collection

The final number of farms included in the study was 38, distributed all over Spain (see Additional file 12: Figure S7). These farms were divided into intensive (19 herds) and extensive (19 herds) farms based on their production system. Farm and sampling characteristics, including sampling season, and antimicrobial consumption of each farm are included in an additional table (see Additional file 13). Sampling was carried out between 2017 and 2018 in feedlots with pigs between 6 and 8 months old. No antimicrobial treatment was administered in the immediate month prior to the sampling.

On each farm, feces, environmental swabs, and slurry were collected. Five fresh fecal samples were obtained from the rectum of fattening pigs. Five samples were swabbed in the environment of the fattening unit (feeders, drinkers, floors, walls, and windows) using swabs soaked in phosphate buffer saline (PBS) 1×. On those farms with slurry pits, three samples were collected from different points of the pit. Nine extensive farms did not provide slurry samples due to the lack of slurry pits in their facilities. The samplings were carried out by trained veterinarians and the samples were sent to our laboratory under cooling conditions (2–8 °C) in less than 24 h.

### Antimicrobial use (AMU)

The veterinary practitioner responsible for each farm recorded the antimicrobial consumption of the pigs of the fattening unit sampled over the immediate 4-month period prior to sampling. AMU was categorized into 10 classes: (i) total, (ii) aminoglycosides, (iii) beta-lactams, (iv) diaminopyrimidines, (v) MLP, (vi) phenicols, (vii) polymyxins, (viii) quinolones, (ix) sulfonamides, and (x) tetracyclines. For each antimicrobial class, consumption per farm was expressed in annual mg/PCU, following the European Surveillance of Veterinary Antimicrobial Consumption (ESVAC) protocol [52].

### Sample processing, DNA extraction, library preparation, and sequencing

From each farm, a single DNA sample was obtained from fecal, environmental and, if available, slurry samples. A total of 105 DNA samples were sequenced from these 38 independent herds, including three DNA extraction negative controls. The samples were divided into environment (38), feces (38), and slurry (29). Prior to DNA extraction, fecal samples from each farm were pooled by stirring thoroughly with a sterile tongue depressor using 3 g per individual sample, obtaining a final composite sample of 15 g. After its homogenization, 2 g were soaked in 18 ml of PBS 1× and vigorously mixed for 5 min using a Stomacher Laboratory Blender (Seward, Worthing, UK). For the environmental and slurry samples, 2 ml were recovered from each individual sample and added to a 15-ml sterile Falcon tube (BD, Erembodegem, Belgium), obtaining a final volume of 10 ml and 6 ml, respectively. These tubes were centrifuged at 4500×*g* for 10 min at 4 °C, discharging the supernatant. Sample handling was performed on ice.

DNA was extracted using the Stool DNA Purification Kit (EUR_X_, Gdańsk, Poland) following the manufacturer’s instructions with minor modifications. As starting material for DNA extractions, 500 μl were used for the three different samples. The final DNA was eluted in 200 μl of 10 mM Tris HCl buffer (pH 8) after its incubation for 5 min for maximum elution efficiency and stored at – 80 °C until its use. Negative controls were prepared with 500 μl of sterile distilled water as starting material and included in DNA extraction batches to confirm that no contamination occurred in the samples during DNA extraction and sequencing.

Prior to sequencing, a Qubit High Sensitivity DNA assay (BioSciences, Dublin, Ireland) was used to determine the total DNA concentration, being its purity assessed by the 260/280 and 260/230 absorbance ratios using a spectrophotometer NanoDrop ND-1000 (Thermo Fisher Scientific, Wilmintong, Delaware). Paired-end sequencing libraries were prepared from the extracted DNA using the Illumina Nextera XTLibrary Preparation Kit (Illumina Inc., San Diego, CA, USA) followed by sequencing on the Illumina NextSeq 500, with a NextSeq 500/550 High Output Reagent kit v2 (300 cycles), in accordance with the standard Illumina sequencing protocols.

### Reads quality filtering

Pre-processing of raw reads by sequence quality and length was performed with PRINSEQ-Lite v0.20.4 [53]. A mean quality lower than Q25 in a 10 base pair sliding window was the criteria utilized for trimming low quality reads at the 3′-end. Moreover, a minimum length of 150 base pairs was ensured for all reads. The Illumina sequences clean were screened against the pig reference genome (*Sus scrofa* UCSC) downloaded from Illumina iGenomes (https://support.illumina.com/sequencing/sequencing_software/igenome.html, 2019) to remove host reads using BMTagger v3.101 (ftp://ftp.ncbi.nlm.nih.gov/pub/agarwala/bmtagger/, 2011). Read duplicates were removed using the Picard MarkDuplicates tool v2.18.1 (https://broadinstitute.github.io/picard/, 2016) to create fastq files with unique reads only. Afterwards, reads were subjected to a further quality filtering step. In brief, sequences were trimmed for low quality score using a modified version of the script trimBWAstyle.pl that works directly from BAM files (TrimBWAstyle.usingBam.pl, 2010; https://github.com/genome/genome/blob/master/lib/perl/Genome/Site/TGI/Hmp/HmpSraProcess/trimBWAstyle.usingBam.pl). The script was used to trim off bases with a quality value of 3 or lower. This threshold was chosen to delete all the bases with an uncertain quality as defined by Illumina’s EAMMS (End Anchored Max Scoring Segments) filter. Additionally, reads trimmed to less than 200bp were also removed.

### Assembly into contigs and taxonomic annotation of reads and contigs

Filtered reads were assembled using IDBA_UD v1.1.3 (kmers 20–120) [54], keeping those contigs with length above 500 bp. Contigs and filtered reads were taxonomically assigned by using Kraken2 software v2.0.8-beta [55] and kraken2-microbial database (2018-09-03) (https://lomanlab.github.io/mockcommunity/mc_databases.html). Only those taxa belonging to the kingdom Bacteria were used for further analyses. The same procedure and database were employed for the taxonomical assignment of both reads and contigs in order to avoid biases caused by the use of different approaches. The relative abundance matrix of filtered reads at family level was used for bacterial microbiome characterization analyses.

### ARGs annotation

Reads from each sample were mapped against the ResFinder database (2019-08-28) [56] using Bowtie2 v2.3.4.1 [57, 58]. The “.trimmed_pairs” fastq files generated by Bowtie2 were transformed into a fasta file where forward and reverse reads were concatenated. This new fasta file was employed to perform a BLAST v2.6.0 [59] against the ResFinder database [56] using a 70% identity cut-off and taking 100 hits (max_target_seqs) in order to avoid problems associated to BLAST use in local [60]. Only the first hit per sequence was kept for further analyses.

The document “phenotypes.txt” was downloaded from the ResFinder repository (2019-10-01) (https://bitbucket.org/genomicepidemiology/resfinder_db/src/master/) and manually curated in order to modify the “class” variable, gathering genes that confer resistance to macrolides, lincosamides, streptogramins, and pleuromutilins into the MLSP class, and those that confer resistance to oxazolidinones, as the oxazolidinone class. This last group included *cfr* genes, which confer resistance to phenicols, lincosamides, oxazolidinones, pleuromutilins and streptogramins A, the *optrA* gene, which confers resistance to phenicols and oxazolidinones, and the *poxtA* gene, which confers resistance to phenicols, oxazolidinones, and tetracyclines.

The manually curated version of phenotypes.txt file (see Additional file 14) was used to create two different matrices from BLASTn-firsthit file: (a) gene abundance, (b) antimicrobial resistance class abundance. Abundance matrices were transformed to CPM matrices, defined as a normalization which consists of scaling the counts by the total number of filtered reads, for further analyses.

### Taxonomical assignment of ARGs

The “.trimmed_pairs” fastq files generated by mapping the reads on the ResFinder database [56] by Bowtie2 [57, 58] were re-mapped against contigs using the same approach, in order to know which ARG-read belonged to each contig.

Taxonomic assignment for each contig was exported to the contained ARG-read, prior to the final quantification of ARG gene and AMR class per taxonomic group at family level. Abundance matrices were transformed to CPM matrices for further analyses.

### AMR mobilome characterization

BLASTn comparison [59] against the ResFinder database [56] was carried out for contigs longer than 1500bp, keeping only those contigs containing ARG for their mobilome analysis. Plasmid location was predicted by PlasFlow v1.1 [61], LGT events were detected by WAAFLE (https://huttenhower.sph.harvard.edu/waafle) and integrons were predicted by Integron_Finder v2 [62], using the assembled contig files as query files.

The coding sequences (CDS) within LGT events and integron regions were extracted by using their coordinates in the contig from WAAFLE and Integron_Finder output files, and bedtools utilities v2.29.0 (https://bedtools.readthedocs.io/en/latest/) to extract this CDS fasta files from contig fasta files. CDS fasta files were used for BLASTn comparison [59] against the ResFinder database [56] to determine which LGT events and integrons contained ARGs. Additionally, complete integron sequences were extracted from contig fasta files and clustered using VSEARCH v2.7.1 [63] with “--cluster_fast” option, to evaluate which integrons were shared between samples.

An in-house ruby script was developed to summarize AMR mobilome outputs in a contig-count per sample matrix, gathering ARGs with chromosomal, plasmid or unknown location, together with LGT events and integron location data. ARGs with unknown location were excluded from further mobilome analyses.

### Statistical analyses and figures visualization

Along the study, analyses were carried out initially for the whole sample collection and later among the two production systems (extensive and intensive) and the three different sample types (environment, feces, and slurry), individually and nested. Besides, the analyses were split into the 16 antimicrobial classes provided by the curated ResFinder database: (i) total, (ii) aminoglycosides, (iii) beta-lactams, (iv) diaminopyrimidines, (v) fosfomycin, (vi) glycopeptides, (vii) MLSP, (viii) multidrug, (ix) nitroimidazoles, (x) phenicols, (xi) oxazolidinones, (xii) polymyxins, (xiii) quinolones, (xiv) rifamycins, (xv) sulfonamides, and (xvi) tetracyclines. All analyses were carried out using R v3.6.2 [64].

The within-herd resistome diversity was computed at the gene-level CPM matrix using the R package vegan v2.5.6 [65]. Alpha diversity was estimated by the Inverse Simpson Diversity (1/D), Simpson (1-D), Shannon and ARGs richness indexes. Comparisons in alpha diversity estimates were carried out with the Wilcoxon signed-rank test through the ggpubr package v0.4.0 [66]. Beta diversity was estimated by Bray-Curtis dissimilarities and analyzed by non-metric multidimensional scaling (NMDS) using the “*metaMDS*” function in vegan. Within-group dispersion was evaluated through the “*betadisper*” function. Finally, the effect of the type of sample and the production system on sample dissimilarities was determined by permutational multivariate analysis of variance (PERMANOVA) using distance matrices with “*adonis2*” function (pairwise adonis**)**. These analyses were also computed for bacterial microbiome characterization at the family-level relative abundance matrix.

Associations among the variables under study (production system and sample type) with the CPM matrices for ARGs and AMR classes, as those taxonomically assigned, the contig-counts matrix for AMR mobilome and the relative abundance matrix for microbiome characterization, were performed with the Kruskal Wallis test and the post hoc Wilcoxon signed-rank test. All *p* values were adjusted by following the Benjamini and Hochberg method [67] and significance was established at *p* < 0.05.

Procrustes analyses were used to determine the association between the resistome and the bacterial microbiome composition. Bray-Curtis dissimilarities from each matrix were ordinated using NMDS. The symmetric Procrustes correlation coefficients between the resistome and the microbiome ordinations, *p* values and plots were obtained using the “*protest*” function in vegan.

As AMU data were strongly skewed, they were log_10_ transformed. In addition, a pseudocount of 1 was added before the log_10_ transformation to deal with an excess of zeros in the data [43]. To reveal the association between AMU and AMR, as the link between AMU and AMR mobilome, the pairwise Spearman’s rank correlation was calculated for CPM matrices for AMR classes and the contig-count AMR mobilome matrix, respectively. Correlations were removed if the Spearman correlation coefficient, *r*_*s*_, was lower than 0.4 and the *p* value > 0.05, adjusting this *p* value to avoid false positives using the Benjamini and Hochberg method [67]. The correlations were carried out with the R package Hmisc v4.4.0 [68].

The circular Bray-Curtis resistome dendrogram was constructed by exporting the dendrogram in Newick format using the R package ape v5.4 [69] and further annotating it using the Interactive Tree of Life tool (https://itol.embl.de/). Other plots were produced using the ggplot2 package v3.3.2 [70], and further modified using the software Inkscape v0.92.4 (https://inkscape.org/). The level of statistical significance was represented with asterisks: four asterisks (****) indicated a *p* value less than 0.0001; three asterisks (***) indicated a *p* value between 0.0001 and 0.001; two asterisks (**) indicated a *p* value between 0.001 and 0.01; one asterisk (*) indicated a *p-*value between 0.01 and 0.05; non-significance (ns) indicated a *p* value higher than 0.05.

## Supplementary Information

**Additional file 1: Figure S1.** Alpha diversity of different antimicrobial resistance (AMR) classes measured by A) Simpson and B) Shannon indexes. These indexes were calculated from the counts per million matrix and represented as boxplots. Each sample is represented by a dot with horizontal jitter for visibility. The horizontal box lines represent the first quartile, the median, and the third quartile. Whiskers include the range of points within the 1.5 interquartile range. The differences per sample type and per production system within each sample type were evaluated with the Wilcoxon signed-rank test. *n* = 105 metagenomes from 38 independent farms. Nineteen metagenomes per sample type per production system were used, with the exception of extensive-slurry (*n* = 9). MLSP refers to the macrolides-lincosamides-streptogramins-pleuromutilins AMR class.

**Additional file 2: Figure S2.** Resistome variation among different types of production system and samples at antimicrobial resistance (AMR) class level. Two-dimension non-metric multidimensional scaling (NMDS) based on Bray-Curtis dissimilarities was calculated for A) Beta-lactams, B) Tetracyclines, C) MLSP and D) Aminoglycosides. Subsampling was carried out by the three types of samples within each production system prior to performing ordination analysis and PERMANOVA. The centroid of each ellipse represents the group mean, and the shape was defined by the covariance within each group. Each NMDS was divided by the two production systems to observe clearer differences. *n* = 105 metagenomes from 38 independent farms. Nineteen metagenomes per production system per sample type were used, with the exception of extensive-slurry (*n* = 9). MLSP refers to the macrolides-lincosamides-streptogramins-pleuromutilins AMR class.

**Additional file 3: Table S1.** Resistome variation among different production systems and samples at AMR class level.

**Additional file 4: Figure S3.** Overview of antimicrobial resistance genes (ARGs) abundance within antimicrobial resistance (AMR) classes per sample. Boxplots of the ARGs in counts per million within each AMR class per sample, were stratified by production system and sample type. Each sample is represented by a dot with horizontal jitter for visibility. The horizontal box lines represent the first quartile, the median, and the third quartile. Whiskers include the range of points within the 1.5 interquartile range. The differences per sample type and per production system within each sample type were evaluated with the Wilcoxon signed-rank test. *n* = 105 metagenomes from 38 independent farms. Nineteen metagenomes per production system per sample type were used, with the exception of extensive-slurry (*n* = 9). MLSP refers to the macrolides-lincosamides-streptogramins-pleuromutilins AMR class.

**Additional file 5: Tables S2-S11.** Significant associations at antimicrobial resistance class and antimicrobial resistance genes level (*p <* 0.05) across production systems and sample types.

**Additional file 6: Figure S4.** Association between the resistome and the bacterial microbiome composition. A) Correlation between antimicrobial resistance genes and bacterial abundance at family level using Procrustes analyses. The lines show the Procrustes residuals; the change in the ordination position when using the resistome (dotted ends) compared to the bacterial microbiome (non-dotted ends) is displayed. The correlation coefficient and significance were determined using the “*protest*” function in R package vegan. B) Residual error plot for Procrustes residual size comparison showing the difference in the resistome-microbiome association across production systems and sample types. Horizontal lines denote the median (solid), 25% and 75% quantiles (dashed). *n* = 105 metagenomes from 38 independent farms. Nineteen metagenomes per production system per sample type were used, with the exception of extensive-slurry (*n* = 9).

**Additional file 7:.** Supplementary information. Supplementary text with additional information.

**Additional file 8: Figure S5.** Bacterial microbiome composition at family level. A) Alpha diversity of bacterial composition measured by family richness, Inverse Simpson, Shannon and Simpson indexes. These indexes were calculated from the relative abundance matrix and represented as boxplots. Each sample is represented by a dot with horizontal jitter for visibility. The horizontal box lines represent the first quartile, the median, and the third quartile. Whiskers include the range of points within the 1.5 interquartile range. The differences per sample type and per production system within each sample type were evaluated with the Wilcoxon signed-rank test. B) Two-dimension non-metric multidimensional scaling (NMDS) based on Bray-Curtis dissimilarities. Subsampling was carried out by the three types of samples within each production system prior to performing ordination analysis and PERMANOVA. The centroid of each ellipse represents the group mean, and the shape was defined by the covariance within each group. C) Stacked bar plot of the relative abundance of the 20 most abundant bacterial families (colors), per sample (x axis); the less abundant families were grouped into “Others”. *n* = 105 metagenomes from 38 independent farms. Nineteen metagenomes per production system per sample type were used, with the exception of extensive-slurry (*n* = 9).

**Additional file 9: Tables S12-S26.** Significant associations at antimicrobial resistance class and antimicrobial resistance genes (ARGs) level (*p <* 0.05) across production systems and sample types in taxonomically assigned ARGs.

**Additional file 10: Tables S27-S28.** Summary of integrons and lateral gene transfer events detected on contigs with more than 1,500bp.

**Additional file 11: Figure S6.** Association between antimicrobial use (AMU) and antimicrobial resistance (AMR). To reveal the association between AMU and AMR, the pairwise Spearman’s rank correlation was calculated for the counts per million matrices at AMR class level. These correlations were carried out for A) all the samples, B) environmental samples, C) faecal samples and D) slurry samples. Correlations were removed if the Spearman correlation coefficient, *r*_*s*_, was lower than 0.4 and the *p*-value > 0.05, adjusting this *p-*value to avoid false positives using the Benjamini & Hochberg method. *n* = 105 metagenomes from 38 independent farms. Nineteen metagenomes per sample type per production system were used, with the exception of extensive-slurry (*n* = 9). MLSP refers to the macrolides-lincosamides-streptogramins-pleuromutilins AMR class. MLP refers to macrolides-lincosamides-pleuromutilins.

**Additional file 12: Figure S7.** Distribution of the 38 Spanish pig farms sampled throughout Spain grouped by their production system into intensive and extensive.

**Additional file 13: Table S29.** Characteristics of 38 independent Spanish pig farms included in the study.

**Additional file 14: Table S30.** Manually curated "phenotypes.txt" from the ResFinder repository.

## Data Availability

DNA sequences from the 105 metagenomic samples from 38 herds and 3 DNA extraction controls are publicly available at the Sequence Read Archive database—NCBI (PRJNA628671).

## Abbreviations

AMR: Antimicrobial resistance
AMU: Antimicrobial use
ARG: Antimicrobial resistance gene
CDS: Coding sequences
CPM: Counts per million
LGT: Lateral gene transfer
MGE: Mobile genetic element
MLSP: Macrolides-lincosamides-streptogramins-pleuromutilins
MLP: Macrolides-lincosamides-pleuromutilins
NMDS: Non-metric multidimensional scaling
PERMANOVA: Permutational multivariate analysis of variance
