# Supplementary figures and images for: Antimicrobial use and production system shape the fecal, environmental, and slurry resistomes of pig farms
Source: Microbiome. 2020 Nov 19;8:164. doi: 10.1186/s40168-020-00941-7 (PMC7678069; doi:10.1186/s40168-020-00941-7)

A)

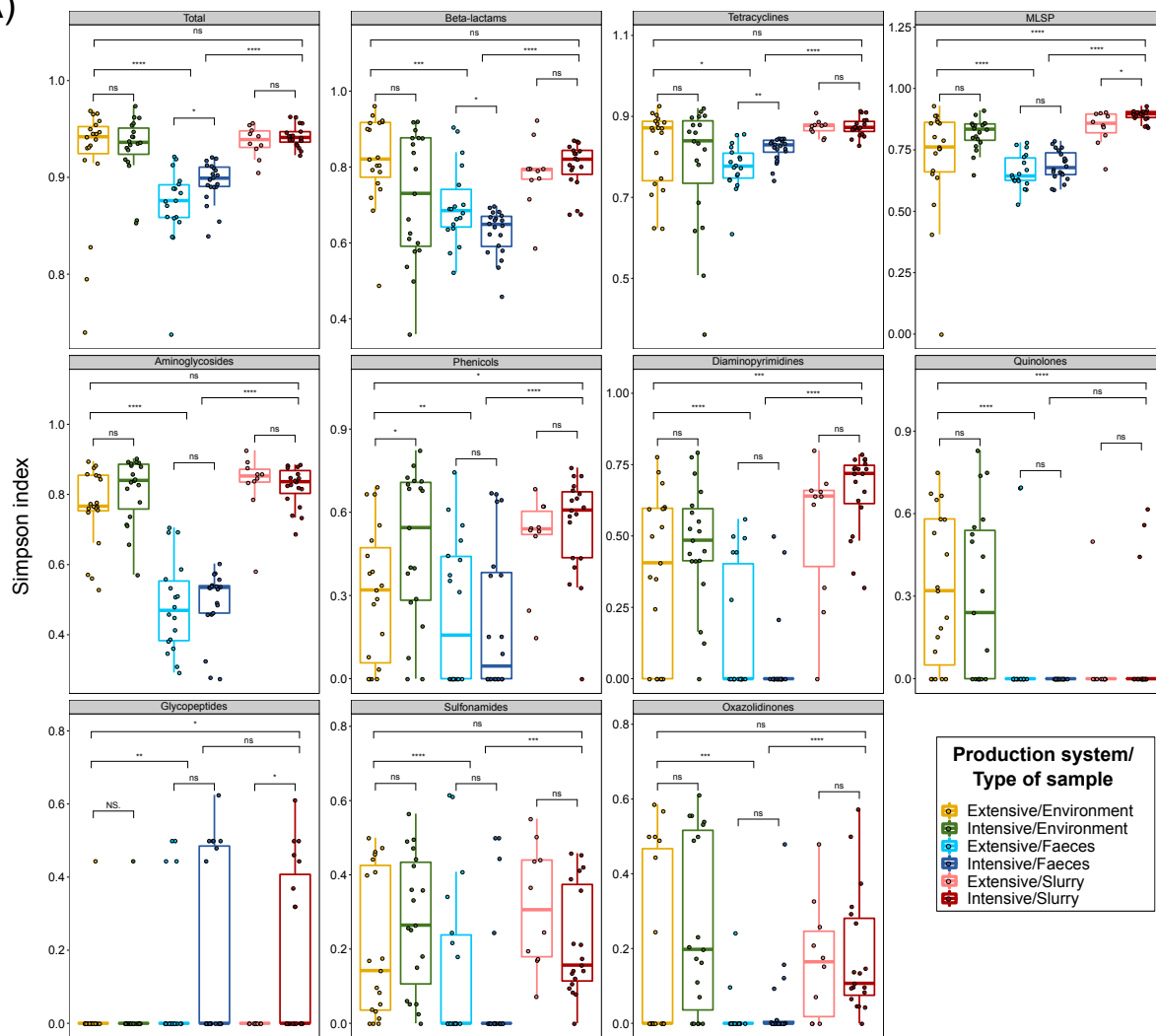

B)

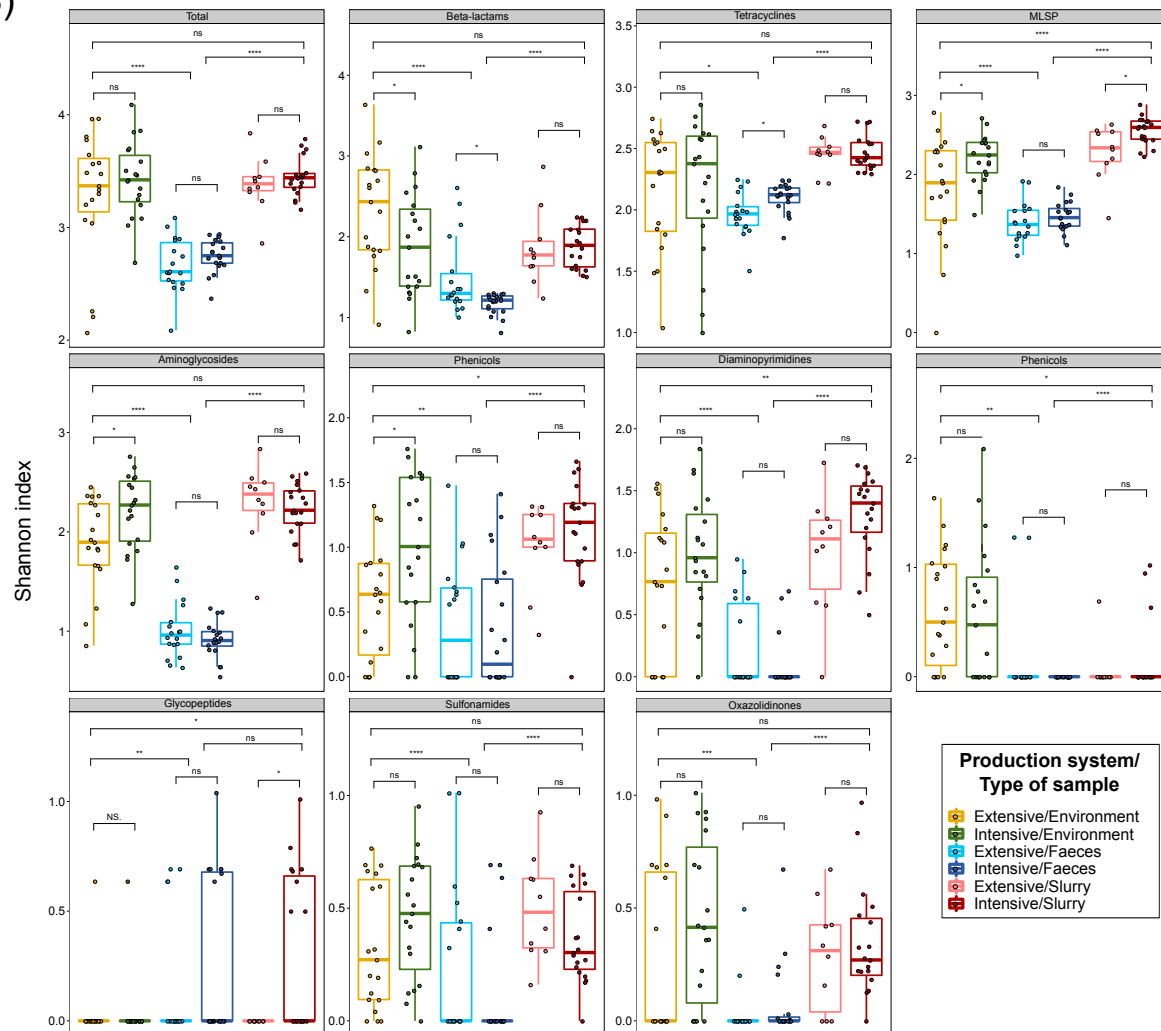

Supplement: Supplementary file 2 — Additional file 1: Figure S1. Alpha diversity of different antimicrobial resistance (AMR) classes measured by A) Simpson and B) Shannon indexes. These indexes were calculated from the counts per million matrix and represented as boxplots. Each sample is represented by a dot with horizontal jitter for visibility. The horizontal box lines represent the first quartile, the median, and the third quartile. Whiskers include the range of points within the 1.5 interquartile range. The differences per sample type and per production system within each sample type were evaluated with the Wilcoxon signed-rank test. n = 105 metagenomes from 38 independent farms. Nineteen metagenomes per sample type per production system were used, with the exception of extensive-slurry (n = 9). MLSP refers to the macrolides-lincosamides-streptogramins-pleuromutilins AMR class. [file 40168_2020_941_MOESM1_ESM.pdf]

A)

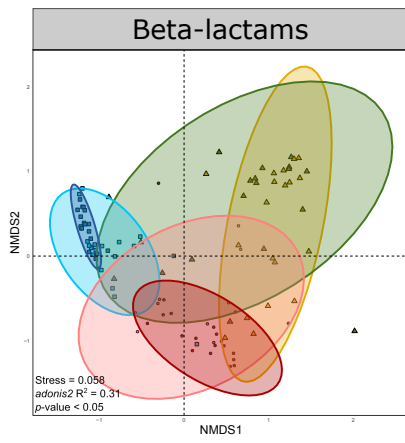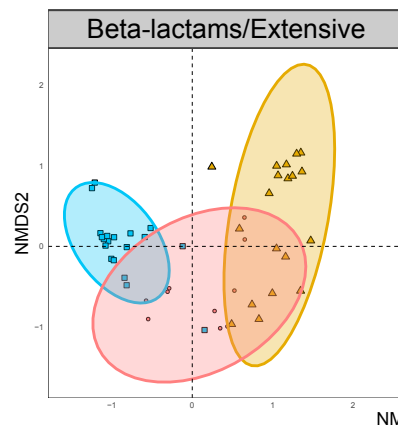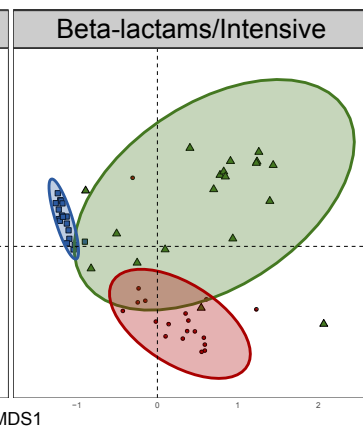

B)

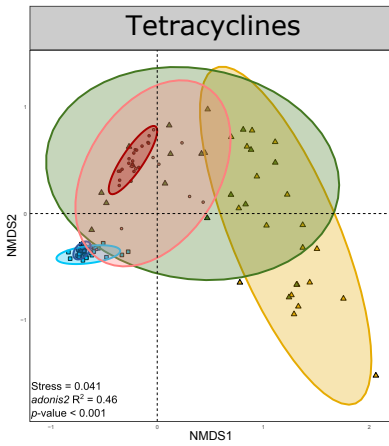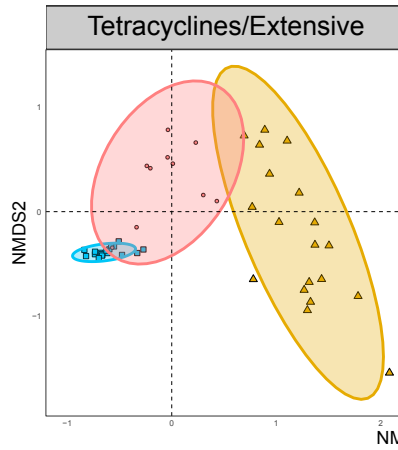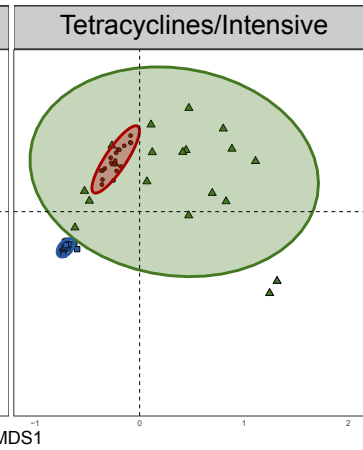

C)

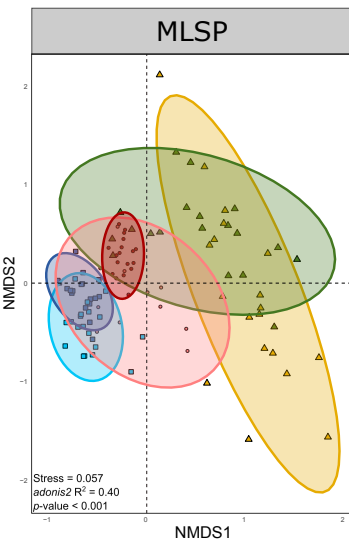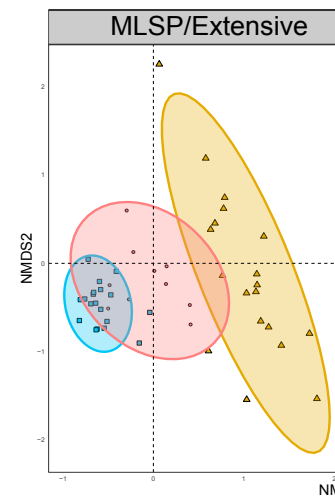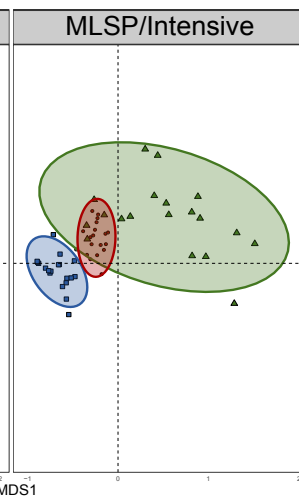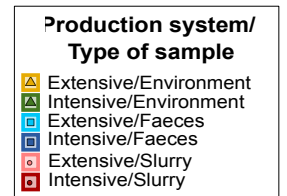

D)

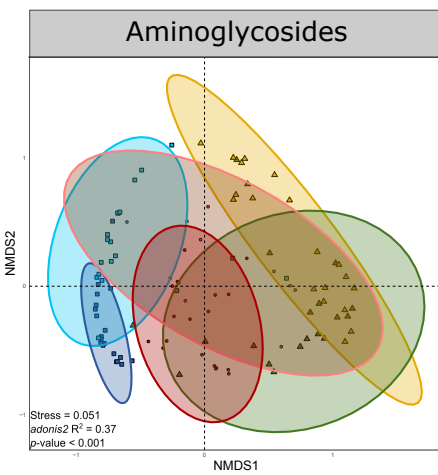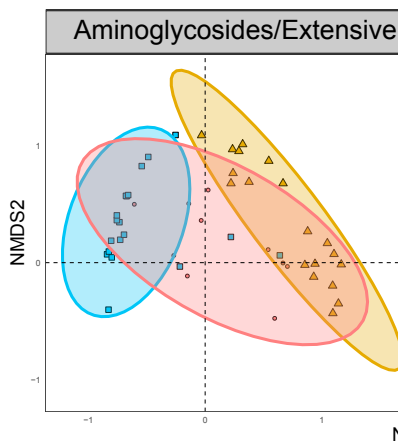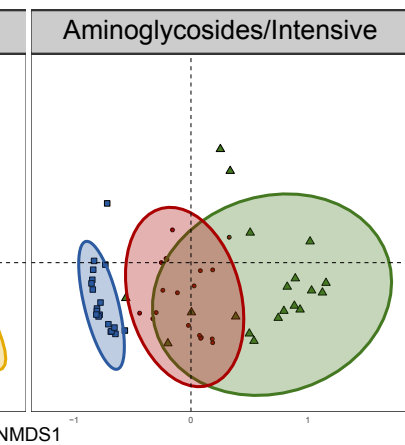

Supplement: Supplementary file 3 — Additional file 2: Figure S2. Resistome variation among different types of production system and samples at antimicrobial resistance (AMR) class level. Two-dimension non-metric multidimensional scaling (NMDS) based on Bray-Curtis dissimilarities was calculated for A) Beta-lactams, B) Tetracyclines, C) MLSP and D) Aminoglycosides. Subsampling was carried out by the three types of samples within each production system prior to performing ordination analysis and PERMANOVA. The centroid of each ellipse represents the group mean, and the shape was defined by the covariance within each group. Each NMDS was divided by the two production systems to observe clearer differences. n = 105 metagenomes from 38 independent farms. Nineteen metagenomes per production system per sample type were used, with the exception of extensive-slurry (n = 9). MLSP refers to the macrolides-lincosamides-streptogramins-pleuromutilins AMR class. [file 40168_2020_941_MOESM2_ESM.pdf]

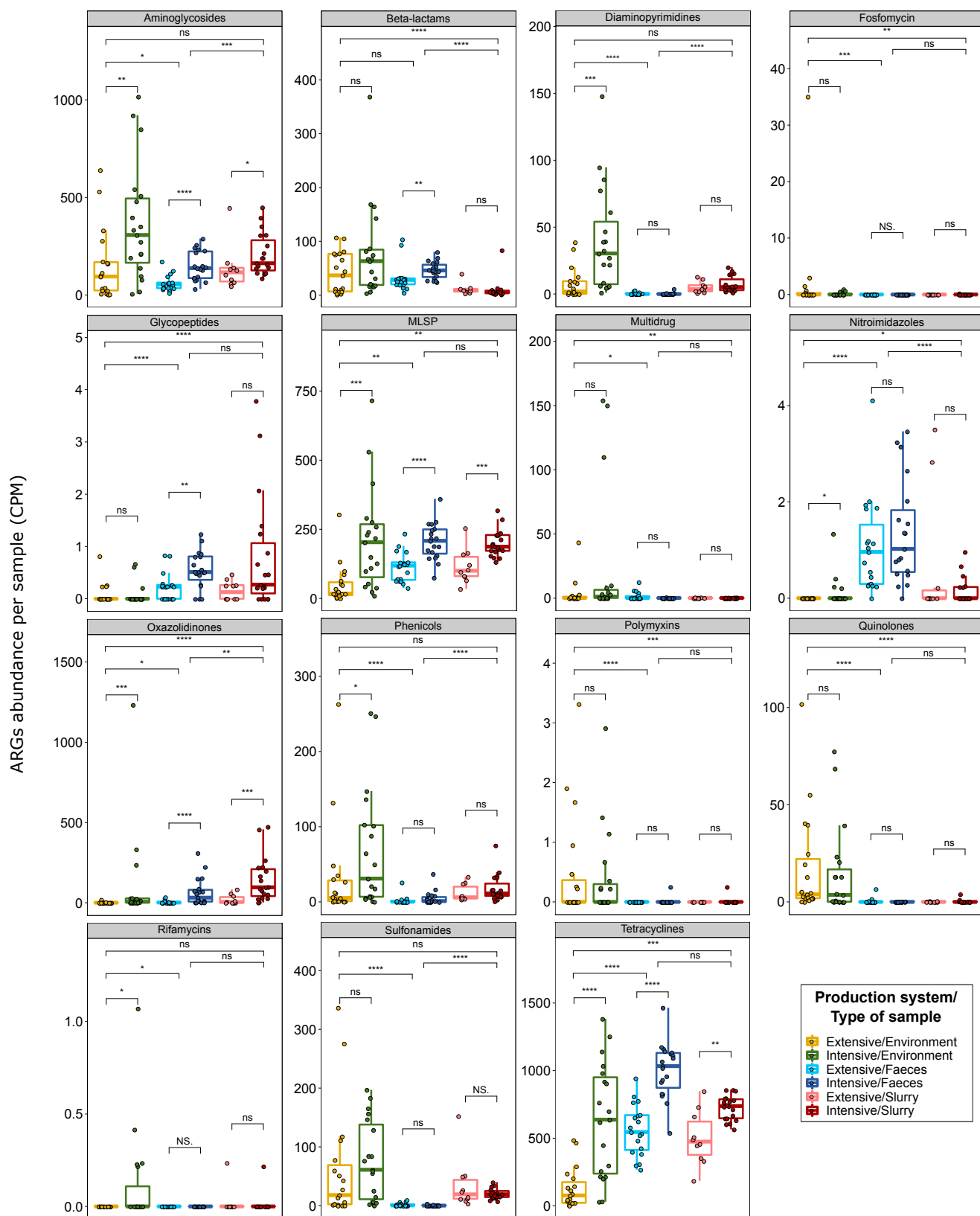

Supplement: Supplementary file 5 — Additional file 4: Figure S3. Overview of antimicrobial resistance genes (ARGs) abundance within antimicrobial resistance (AMR) classes per sample. Boxplots of the ARGs in counts per million within each AMR class per sample, were stratified by production system and sample type. Each sample is represented by a dot with horizontal jitter for visibility. The horizontal box lines represent the first quartile, the median, and the third quartile. Whiskers include the range of points within the 1.5 interquartile range. The differences per sample type and per production system within each sample type were evaluated with the Wilcoxon signed-rank test. n = 105 metagenomes from 38 independent farms. Nineteen metagenomes per production system per sample type were used, with the exception of extensive-slurry (n = 9). MLSP refers to the macrolides-lincosamides-streptogramins-pleuromutilins AMR class. [file 40168_2020_941_MOESM4_ESM.pdf]

A)

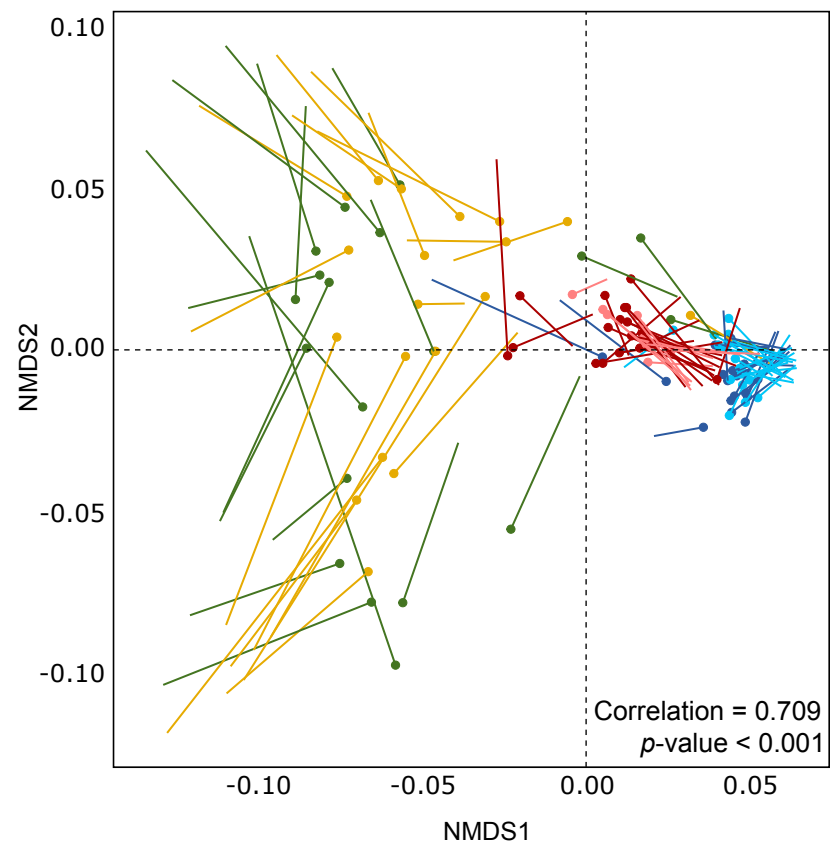

B)

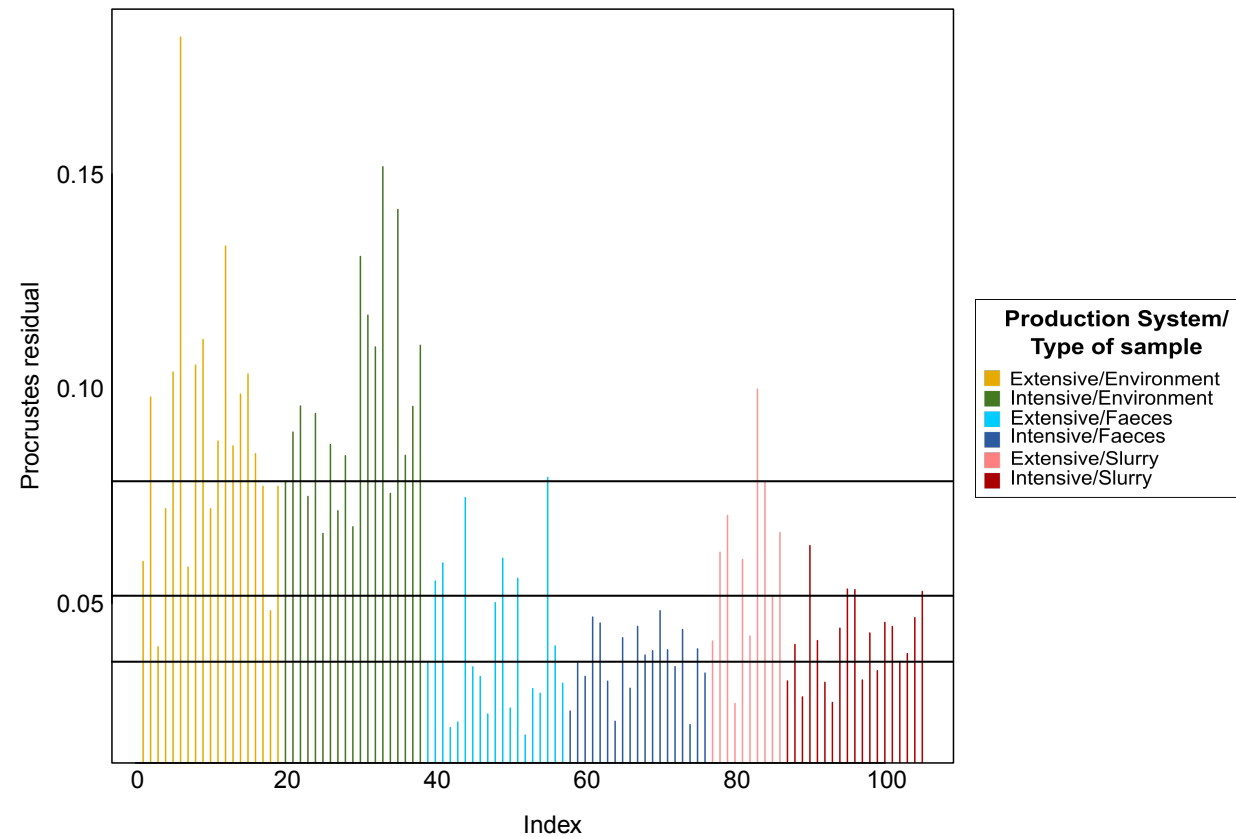

Supplement: Supplementary file 7 — Additional file 6: Figure S4. Association between the resistome and the bacterial microbiome composition. A) Correlation between antimicrobial resistance genes and bacterial abundance at family level using Procrustes analyses. The lines show the Procrustes residuals; the change in the ordination position when using the resistome (dotted ends) compared to the bacterial microbiome (non-dotted ends) is displayed. The correlation coefficient and significance were determined using the “protest” function in R package vegan. B) Residual error plot for Procrustes residual size comparison showing the difference in the resistome-microbiome association across production systems and sample types. Horizontal lines denote the median (solid), 25% and 75% quantiles (dashed). n = 105 metagenomes from 38 independent farms. Nineteen metagenomes per production system per sample type were used, with the exception of extensive-slurry (n = 9). [file 40168_2020_941_MOESM6_ESM.pdf]

A)

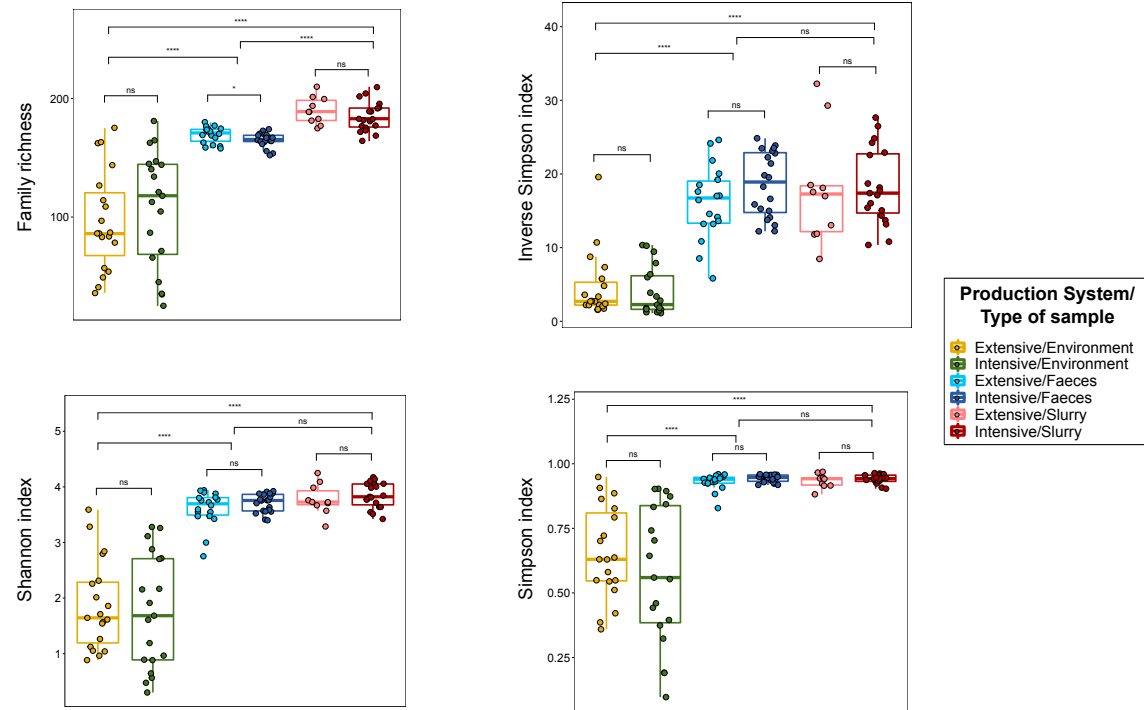

B)

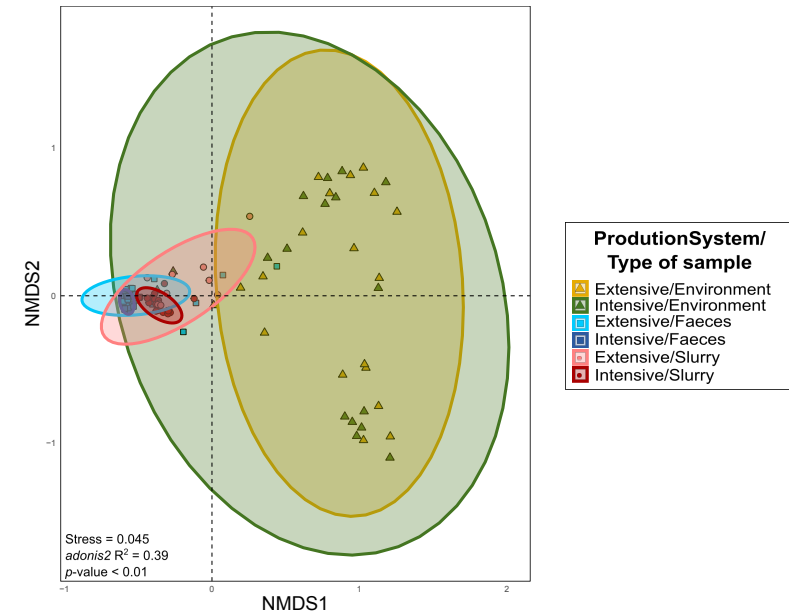

C)

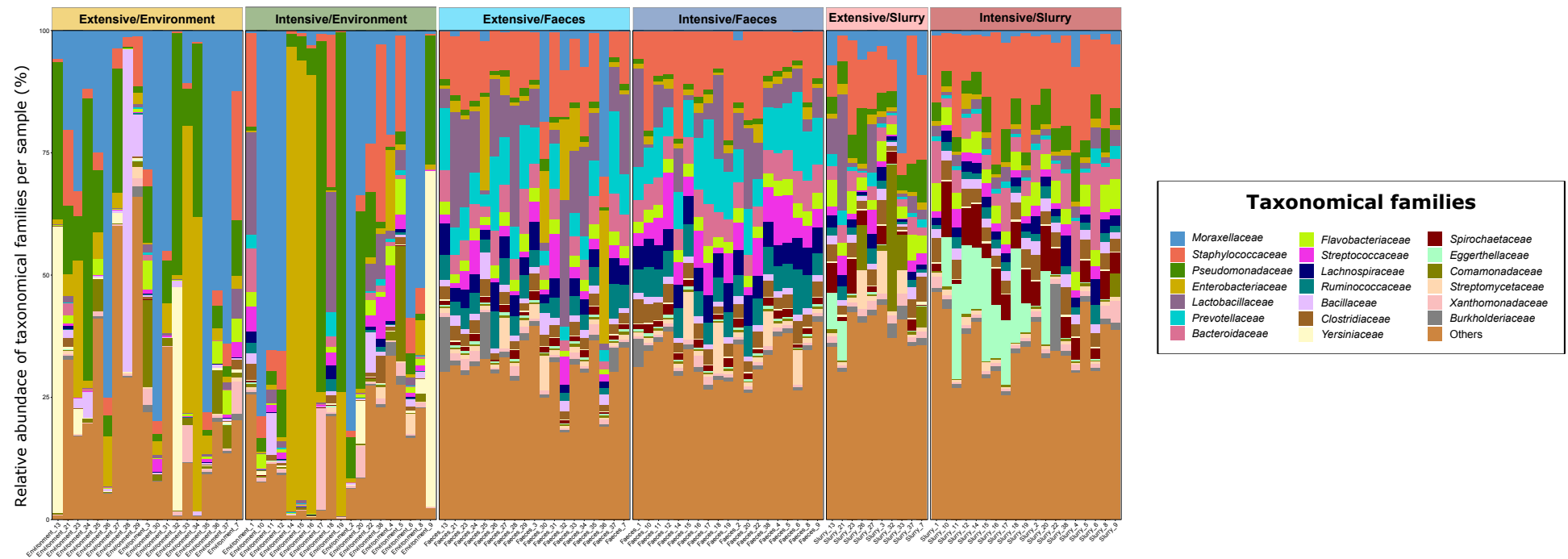

Supplement: Supplementary file 9 — Additional file 8: Figure S5. Bacterial microbiome composition at family level. A) Alpha diversity of bacterial composition measured by family richness, Inverse Simpson, Shannon and Simpson indexes. These indexes were calculated from the relative abundance matrix and represented as boxplots. Each sample is represented by a dot with horizontal jitter for visibility. The horizontal box lines represent the first quartile, the median, and the third quartile. Whiskers include the range of points within the 1.5 interquartile range. The differences per sample type and per production system within each sample type were evaluated with the Wilcoxon signed-rank test. B) Two-dimension non-metric multidimensional scaling (NMDS) based on Bray-Curtis dissimilarities. Subsampling was carried out by the three types of samples within each production system prior to performing ordination analysis and PERMANOVA. The centroid of each ellipse represents the group mean, and the shape was defined by the covariance within each group. C) Stacked bar plot of the relative abundance of the 20 most abundant bacterial families (colors), per sample (x axis); the less abundant families were grouped into “Others”. n = 105 metagenomes from 38 independent farms. Nineteen metagenomes per production system per sample type were used, with the exception of extensive-slurry (n = 9). [file 40168_2020_941_MOESM8_ESM.pdf]

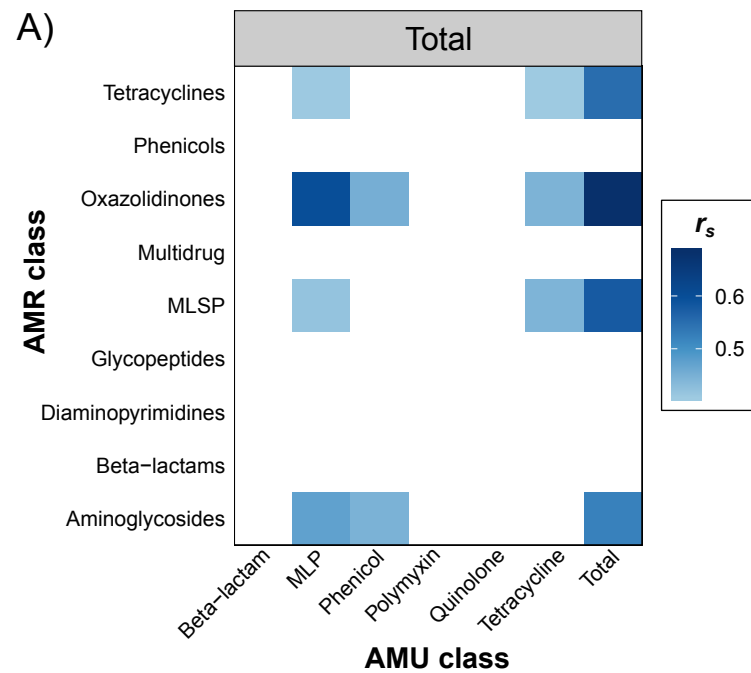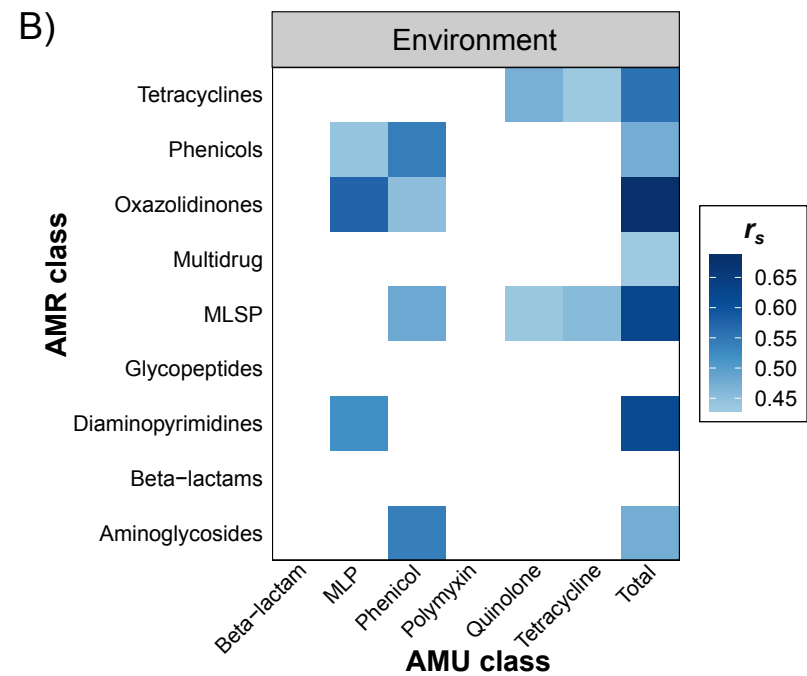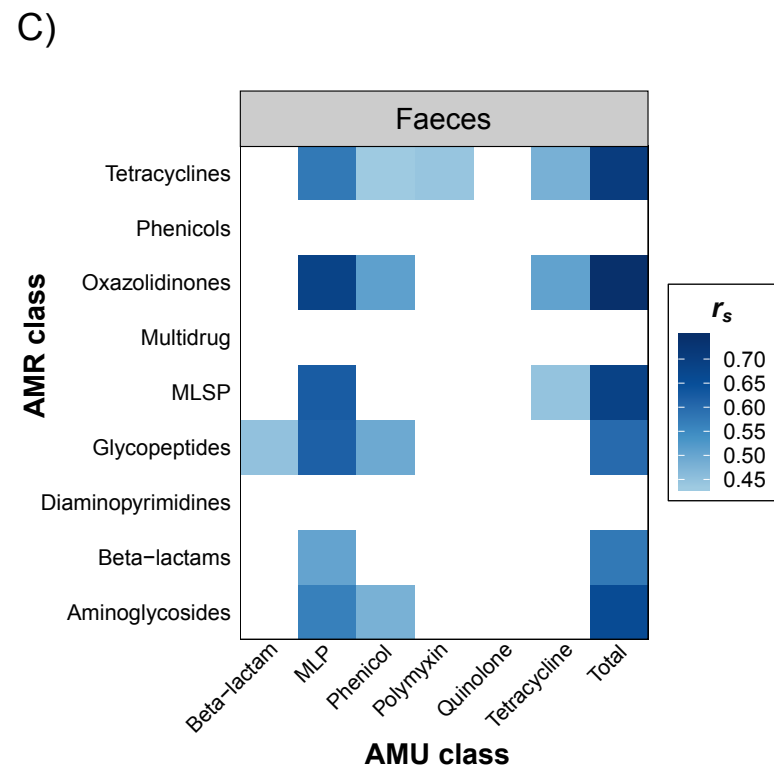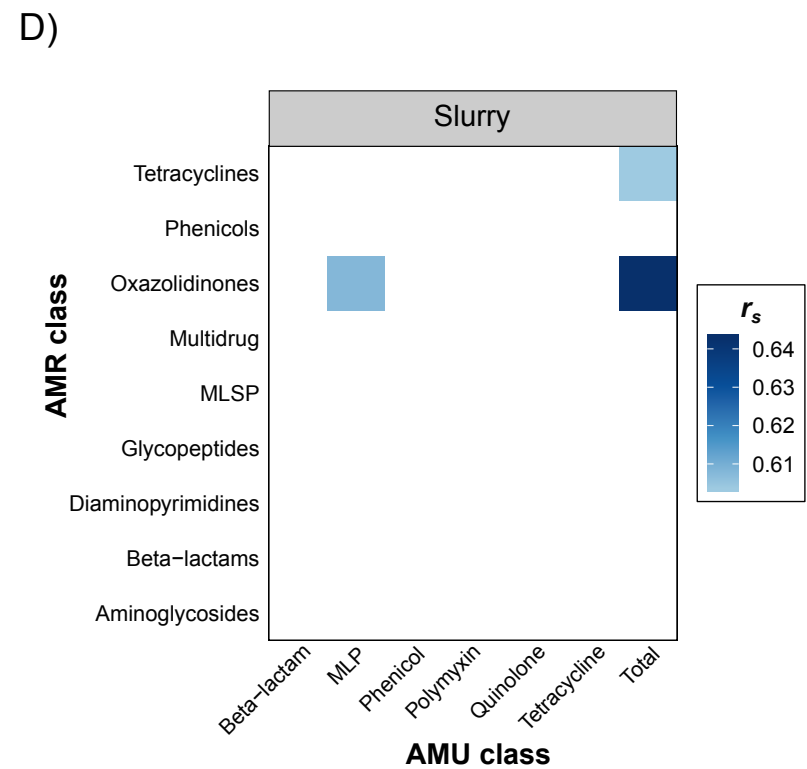

Supplement: Supplementary file 12 — Additional file 11: Figure S6. Association between antimicrobial use (AMU) and antimicrobial resistance (AMR). To reveal the association between AMU and AMR, the pairwise Spearman’s rank correlation was calculated for the counts per million matrices at AMR class level. These correlations were carried out for A) all the samples, B) environmental samples, C) faecal samples and D) slurry samples. Correlations were removed if the Spearman correlation coefficient, rs, was lower than 0.4 and the p-value > 0.05, adjusting this p-value to avoid false positives using the Benjamini & Hochberg method. n = 105 metagenomes from 38 independent farms. Nineteen metagenomes per sample type per production system were used, with the exception of extensive-slurry (n = 9). MLSP refers to the macrolides-lincosamides-streptogramins-pleuromutilins AMR class. MLP refers to macrolides-lincosamides-pleuromutilins. [file 40168_2020_941_MOESM11_ESM.pdf]

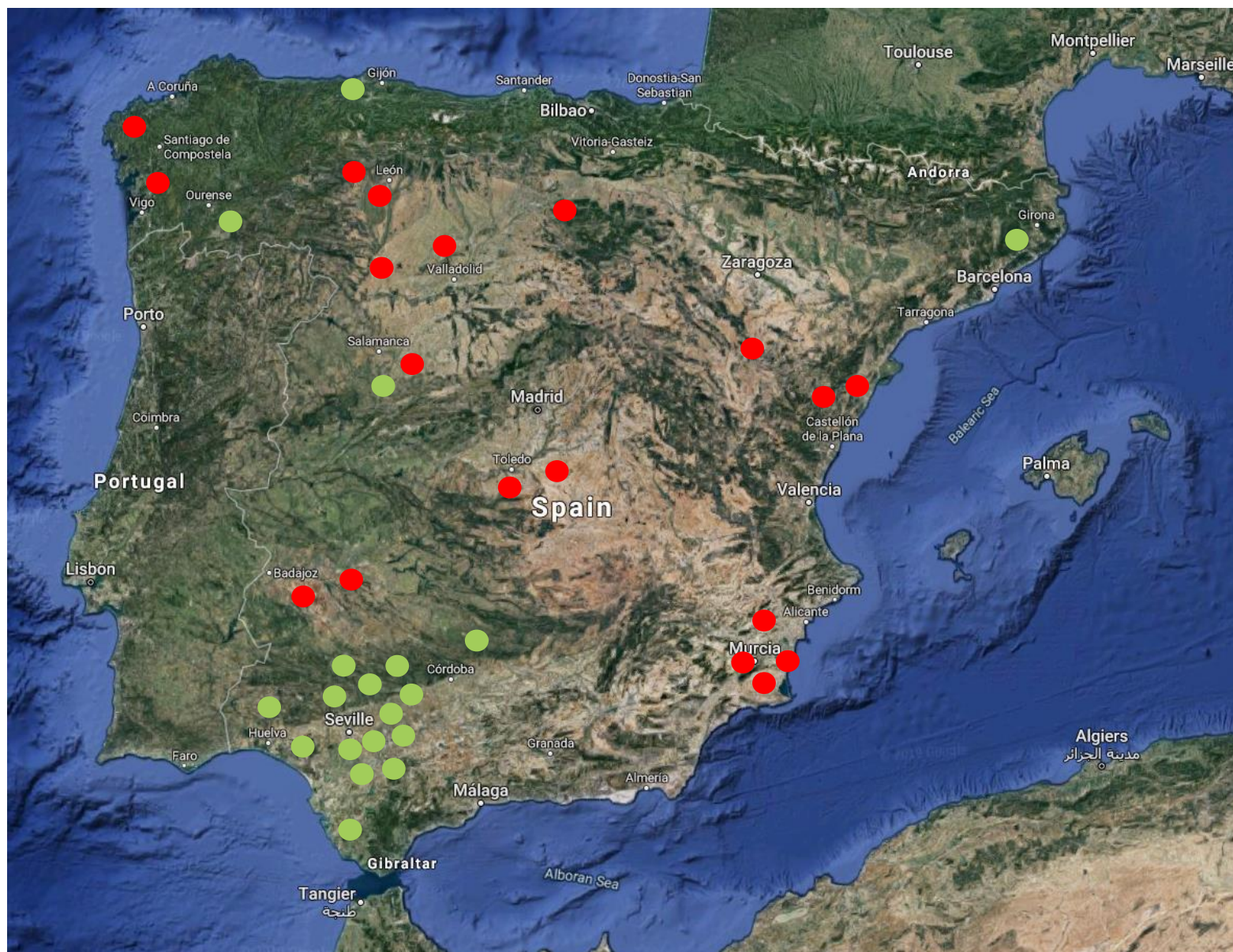

### Production System

- Intensive
- Extensive

Supplement: Supplementary file 13 — Additional file 12: Figure S7. Distribution of the 38 Spanish pig farms sampled throughout Spain grouped by their production system into intensive and extensive. [file 40168_2020_941_MOESM12_ESM.pdf]
