# Supplementary material for: Antimicrobial use and production system shape the fecal, environmental, and slurry resistomes of pig farms
Source: Microbiome. 2020 Nov 19;8:164. doi: 10.1186/s40168-020-00941-7 (PMC7678069; doi:10.1186/s40168-020-00941-7)
Supplement: Supplementary file 4 — Additional file 3: Table S1. Resistome variation among different production systems and samples at AMR class level. [file 40168_2020_941_MOESM3_ESM.docx]

**Additional file 3: Table S1.** Resistome variation among different production systems and samples at AMR class level.

|  |  | **AMR class** | | | |
| --- | --- | --- | --- | --- | --- |
|  |  | **Aminoglycosides** | **Beta-lactams** | **^d^ MLSP** | **Tetracyclines** |
| **^a, c^ Total**  **sampling** | *Production System* | 8.4 | 1.5 | 5.4 | 5.2 |
|  | *Type of sample* | 28.8 | 29.6 | 34.3 | 40.8 |
| **^b, c^ Type of sample** | *Environment* | 8.4 | 5.4 | 8.4 | 11.3 |
|  | *Faeces* | 31.5 | 16.1 | 26.4 | 41.2 |
|  | *Slurry* | 14.3 | 6.4 | 16.8 | 19.0 |

^a^ Evaluation of the effect of the production system (extensive and intensive) and the sample type (environment, faeces and slurry).

^b^ Evaluation of the effect of the production system on each type of.

^c^ Effects were determined with PERMANOVA analysis and expressed as the percentage of variation explained by each factor for significant p-values (*p* < 0.05).

^d^ MLSP refers to macrolides-lincosamides-streptogramins-pleuromutilins AMR class.
