# Supplementary material for: Antimicrobial use and production system shape the fecal, environmental, and slurry resistomes of pig farms
Source: Microbiome. 2020 Nov 19;8:164. doi: 10.1186/s40168-020-00941-7 (PMC7678069; doi:10.1186/s40168-020-00941-7)
Supplement: Supplementary file 8 — Additional file 7:. Supplementary information. Supplementary text with additional information. [file 40168_2020_941_MOESM7_ESM.docx]

**Additional file 7: Supplementary information.** Supplementary text with additional information.

**Supplementary information accompanying Mencía-Ares et al (*Antimicrobial use and production system shape the faecal, environmental and slurry resistomes of pig farms*)**

**Bacterial microbiome composition**

Alpha diversity indexes revealed a significantly lower bacterial diversity at family level on farm environments, both from intensive and extensive herds, compared to faecal and slurry samples. Analyses by production system only showed a significantly higher family richness in faeces from extensive herds than on those from intensive farms (see Additional file 8: Figure S5A).

The beta diversity using the Bray-Curtis dissimilarity index revealed the combined influence of sample type and production system (adonis2, *p* < 0.01) in the taxonomical ordination of samples, as it was observed in the resistome, with a particularly high variability on farm environments (see Additional file 8: Figure S5B). While the type of sample explained 36.8% of the variation, the production system barely explained the 3.7%.

This high variability and low alpha diversity on farm environments were evidenced by the distribution of the 20 most abundant bacterial families, with a predominance of members of *Moraxellaceae*, *Pseudomonadaceae* and *Enterobacteriaceae* families. In contrast, faecal and slurry samples showed a higher homogeneity, with *Staphylococcaceae* as the most prevalent family in both sample types (see Additional file 8: Figure S5C).
