# Supplementary material for: Antimicrobial use and production system shape the fecal, environmental, and slurry resistomes of pig farms
Source: Microbiome. 2020 Nov 19;8:164. doi: 10.1186/s40168-020-00941-7 (PMC7678069; doi:10.1186/s40168-020-00941-7)
Supplement: Supplementary file 14 — Additional file 13: Table S29. Characteristics of 38 independent Spanish pig farms included in the study. [file 40168_2020_941_MOESM13_ESM.docx]

**Additional file 13: Table S29.** Characteristics of 38 independent Spanish pig farms included in the study.

|  |  | **^a^ Production system** | |  |
| --- | --- | --- | --- | --- |
|  |  | **Intensive**  **(n = 19)** | **Extensive**  **(n = 19)** | |
| **Type of farm** | Farrow-to-Finish | 7 | 16 | |
|  | Multi-site production | 12 | 3 | |
| **Type of pig** | Iberian autochthonous breed | 4 | 16 | |
|  | Other autochthonous breeds | 0 | 3 | |
|  | White crossbred pigs | 15 | 0 | |
| **Sampling season** | Winter | 7 | 2 | |
|  | Spring | 6 | 1 | |
|  | Summer | 4 | 10 | |
|  | Autumn | 2 | 6 | |
| **^b^ Fattening pigs** | | 1521.90  (969.42-2074.37) | 418.88  (158.12-679.63) | |
| **^b, c^ Antimicrobial consumption** | Total | 243.76  (372.63-114.88) | 16.29  (0-39.07) | |
|  | ^d^ MLP | 109.36  (3.72-215.00) | 0  (0-3.72) | |
|  | Tetracyclines | 64.08  (23.70-104.47) | 10.42  (0-31.29) | |
|  | Beta-lactams | 40.57  (8.77-72.36) | 3.64  (0.55-6.73) | |
|  | Aminoglycosides | 16.39  (0-39.09) | 0.99  (0-2.56) | |
|  | Polymyxins | 5.65  (0-13.48) | 1.05  (0-3.28) | |
|  | Sulfonamides | 2.31  (0-7.16) | 0 | |
|  | Phenicols | 2.46  (0.09-4.82) | 0 | |
|  | Quinolones | 2.48  (0.99-3.96) | 0.19  (0-0.47) | |
|  | Diaminopyrimidins | 0.46  (0-1.43) | 0 | |

^a^ Farms were categorized according to their production system into intensive and extensive.

^b^ Fattening pigs and antimicrobial consumption are expressed as the mean and the 95% CI of the data provided by the farms within each group.

^c^ Antimicrobial consumption is expressed in annual mg/PCU.

^d^ MLP refers to macrolides-lincosamides-pleuromutilins.
